# Elucidating the callus-to-shoot-forming mechanism in *Capsicum annuum* ‘Dempsey’ through comparative transcriptome analyses

**DOI:** 10.1186/s12870-024-05033-4

**Published:** 2024-05-07

**Authors:** Sang-Yun Han, So Young Park, Kang-Hee Won, Sung-il Park, Jae-Hyeong Park, Donghwan Shim, Inhwan Hwang, Dong-Hoon Jeong, Hyeran Kim

**Affiliations:** 1 Department of Biological Sciences, Institute for Life Sciences, Kangwon National University, Chuncheon, 24341 Korea; 2 Department of Life Science, Multidisciplinary Genome Institute, Hallym University, Chuncheon, 24252 Korea; 3 Department of BIT Medical Convergence, Kangwon National University, Chuncheon, 24341 Korea; 4 Department of Biological Sciences, Chungnam National University, Daejeon, 34134 Korea; 5 Department of Life Sciences, Pohang University of Science and Technology, Pohang, 37673 Korea

**Keywords:** *Capsicum annuum*, Bell pepper ‘Dempsey’, Regeneration, Transcriptome, Shoot formation, Hypoxia, Defense mechanism, Auxin redistribution

## Abstract

**Background:**

The formation of shoots plays a pivotal role in plant organogenesis and productivity. Despite its significance, the underlying molecular mechanism of *de novo* regeneration has not been extensively elucidated in *Capsicum annuum* ‘Dempsey’, a bell pepper cultivar. To address this, we performed a comparative transcriptome analysis focusing on the differential expression in *C. annuum* ‘Dempsey’ shoot, callus, and leaf tissue. We further investigated phytohormone-related biological processes and their interacting genes in the *C. annuum* ‘Dempsey’ transcriptome based on comparative transcriptomic analysis across five species.

**Results:**

We provided a comprehensive view of the gene networks regulating shoot formation on the callus, revealing a strong involvement of hypoxia responses and oxidative stress. Our comparative transcriptome analysis revealed a significant conservation in the increase of gene expression patterns related to auxin and defense mechanisms in both callus and shoot tissues. Consequently, hypoxia response and defense mechanism emerged as critical regulators in callus and shoot formation in *C. annuum* ‘Dempsey’. Current transcriptome data also indicated a substantial decline in gene expression linked to photosynthesis within regenerative tissues, implying a deactivation of the regulatory system governing photosynthesis in *C. annuum* ‘Dempsey’.

**Conclusion:**

Coupled with defense mechanisms, we thus considered spatial redistribution of auxin to play a critical role in the shoot morphogenesis via primordia outgrowth. Our findings shed light on shoot formation mechanisms in *C. annuum* ‘Dempsey’ explants, important information for regeneration programs, and have broader implications for precise molecular breeding in recalcitrant crops.

**Supplementary Information:**

The online version contains supplementary material available at 10.1186/s12870-024-05033-4.

## Introduction

Shoot formation is a central topic in both plant science and agricultural biotechnology. It is important for several key processes, notably the generation of transgenic plants and the propagation of desired plants. From shoot formation on the pluripotent callus, plants reproduce tissues such as stems, roots, and leaves during vegetative propagation; thus, each organ forms a new plant. The success of whole plant regeneration largely depends on understanding when and how shoots develop from the proliferating cells of explants. Shoot formation is a complex process driven by specific genetic networks and molecular pathways of auxin and cytokinin [1–6]. Pluripotency, derived from somatic cells, is pivotal for the formation of all plant organs throughout a plant’s life cycle. Notably, stem cells found in the shoot apical meristem (SAM) and root apical meristem (RAM) play vital roles, with their functions defined by their location. The totipotency of leaf tissues allows somatic embryos to form from individual leaf cells or cell groups, revisiting early developmental stages in mature plants [5, 7]. Therefore, delving into the molecular underpinnings of shoot formation provides rich insights into plant growth, reproduction, survival, and adaptability.

Phytohormone-related genes are critical for SAM development in callus tissue. In in vitro tissue cultures, phytohormones regulate transcription factors such as CUP-SHAPED COTYLEDON (CUC), which responds to auxin and brassinosteroid (BR), and WUSCHEL (WUS), which is activated by high cytokinin and low auxin conditions. Overexpression of these transcription factors leads to somatic embryos for shoot formation on pluripotent callus tissue [3–5, 8]. PIN-FORMED 1 (PIN1), an auxin efflux carrier, is instrumental in new SAM formation by regulating auxin accumulation [1, 4, 5]. The ethylene-related genes ETHYLENE OVERPRODUCER 1 (ETO1) and ETHYLENE RESPONSE 1 (ETR1) also influence shoot formation. This is likely linked to auxin and cytokinin responses, based on explant sensitivity to ethylene signaling [6]. ETHYLENE RESPONSE FACTOR 109 (ERF109) contributes to tissue repair and organ formation by regulating stem cell activity and auxin production [9, 10], and ERF109-mediated responses are tightly controlled by multiple phytohormones like ethylene (ET), abscisic acid (ABA), and jasmonic acid (JA) [11]. Therefore, a crosstalk of phytohormones centered around auxin within a genetic network plays a pivotal role in plant morphogenesis.

Auxin flow is a significant factor in shoot formation and post-embryonic organogenesis, including the formation of new leaves, flowers, and lateral roots. Plant organogenesis, highlighted by local auxin accumulation at the initiation sites of emerging organs, creates distinct phyllotactic patterns essential for organized plant growth [4, 12–14]. The concentrations and ratios of auxin and cytokinin determine specific developmental pathways in the SAM during shoot growth [4–6, 15]. Furthermore, auxin responses are related to the onset of plasmodesmata production in the cell wall, marking a commitment to regeneration [2]. The physiological role of auxin during organ formation also encompasses the differentiation of new vasculature, including leaf venation [13, 16].

Plant organogenesis comprises fine-tuned developmental processes. Pinpointing the core regulatory system for shoot formation during the developmental processes ensures proper and efficient schemes for plant regeneration. A genome-wide association study (GWAS) of shoot formation using 190 natural Arabidopsis accessions reported that a smaller set (∼ 5%) of identified genes serve as master regulators that are crucial under multiple procedures and traits [15]. A comparative transcriptomic study within Solanaceae—including tomato, potato, petunia, pepper, tobacco, and *Nicotiana benthamiana*—showed a high degree of sequence conservation and species-specific transcripts even though these six species represent diverse phenotypes for different agronomic purposes [17]. Both petunia and Arabidopsis are known as representative regenerating species [18], whereas bell pepper is treated as a recalcitrant species. Thus, it is crucial to consolidate the essential genetic factors by considering distinct genetic backgrounds across species.

*Capsicum annuum* ‘Dempsey’ is a sweet and bell pepper having virus- and bacterial spot-resistant traits that originates from a three-way cross between the ‘PI163192′, ‘PI264281′, and ‘Jupiter’ cultivars [19–21]. The ‘Dempsey’ cultivar is highlighted as an excellent genetic resource with multiple disease resistances, a non-functional *pun1* allele as a standard of non-pungency [22], and available whole genomic information [23]. Besides serving as crop feed, bell peppers are also a great source of antioxidants, especially carotenoids; phenolic compounds; and vitamins A, C, and E [20, 21, 24]. ‘Dempsey’ is genetically distinguishable from other cultivars within the sweet pepper group by comparative analysis among plastome sequences [25] and displays different cellular properties in its polyethylene glycol (PEG)-mediated CRISPR/Cas9 ribonucleoprotein (RNP) delivery compared to hot pepper [26, 27]. Various attempts to apply genome-editing tools to ‘Dempsey’ and other peppers have overcome challenges related to genetic transformation, regeneration, and the study of molecular function in pepper [3, 21, 22, 27–29]. However, studying the molecular mechanism of shoot formation, which is essential for pepper functional genetics, precise molecular breeding, and biotechnology applications, is lacking. The knowledge acquired from studying shoot development in bell peppers can lead to pepper genetic manipulation improving quality and increasing yields.

Herein, we first report on comparative transcriptomic analyses of the ‘Dempsey’ cultivar to identify critical shoot-forming genes expressed during plant regeneration. The comparison included contrasting transcriptomes of callus and shoot segments induced from leaf tissue. Next, we aimed to uncover the genetic orchestration underlying shoot development from leaf explants in the ‘Dempsey’ cultivar, particularly focusing on phytohormones, by comparing a wide range of developmental gene expression profiles across five intra- and inter-family species: ‘Dempsey’ pepper, three petunias, and Arabidopsis. We also identify vital genes in callus-to-shoot organogenesis using gene expression profiling with enriched pathways. Our findings elucidate novel components of the shoot-forming mechanism and highlight potential genetic markers that can be instrumental for pepper transformation and molecular breeding, with practical applications in future agricultural biotechnology.

## Materials and methods

### Plant materials

The bell pepper *C. annuum* ‘Dempsey’ was provided by the Vegetable Breeding Research Center (VBRC) in Seoul, Republic of Korea. For RNA isolation and library preparation, 5-week-old or 18-week-old *C. annuum* ‘Dempsey’ fully expanded leaf tissues near the shoot apical bud were used as the WT. ‘Dempsey’ callus and shoot were prepared using the previously described method [21]. For generating callus and shoot tissues, 1 cm of young apical leaves from 5-week-old *C. annuum* ‘Dempsey’ plants were placed on a shoot induction medium (SIM), and the produced tissues were collected after four weeks. The explants were dissected using a surgical scalpel under a stereo microscope, frozen using liquid nitrogen, and finely ground with a mortar and pestle. The resulting finely ground tissue powder was stored at -80℃ for further experiments.

### Total RNA isolation

Total RNA was isolated from ‘Dempsey’ leaf, callus, and shoot tissue using Tri-RNA Reagent (Favorgen, FATRR 001), and RNA concentrations were measured using a NanoDrop 2000 spectrophotometer (Thermo Fisher Scientific, Waltham, MA, USA; ND-2000). Each RNA sample’s quality was checked using a bioanalyzer (Agilent Technologies, Santa Clara, CA, USA; 2100 Bioanalyzer), and the RNA integrity number (RIN) was confirmed to be above 8.

### RNA-seq library preparation

Following the manufacturer’s protocol, RNA-seq libraries were constructed from two biological replicates using a TruSeq Stranded mRNA Library Prep Kit (Illumina, San Diego, CA, USA; RS-122-2101). They were constructed using the indexed adaptors provided in the kit and pooled for sequencing. Sequencing with a paired 2 × 75 bp length was performed on the Illumina MiSeq platform. The paired-end raw sequencing reads were cleaned (satisfying Q20 and Q30 ≥ 80%) and adaptors were trimmed using the CLC Genomics Workbench v20.0.4 (Liu & Di 2020). The quality of trimmed reads was checked using the FastQC program [30]. All cleaned RNA-seq libraries were deposited at NCBI under BioProject accession number PRJNA1063381.

### Mapping of RNA-seq reads and abundance estimation

The clean reads of each RNA-seq library were mapped using the HI-SAT2 program [31] and the *C. annuum* ‘Dempsey’ genome assembly ASM2707356v1 (NCBI accession number: GCA_027073565.1) [23]. The ‘Dempsey’ transcripts were annotated by running the BLAST program with the transcripts of *Petunia axillaris*, *Petunia exserta*, and *Petunia integrifolia*, and the NCBI datasets of *C. annuum* ‘Zunla-1’ (accession number GCF_000710875.1), *C. annuum* ‘UCD-10X-F1’ (accession number GCF_002878395.1), *Capsicum baccatum* ‘PBC81’ (accession number GCA_002271885.2), and *A. thaliana* TAIR10.1 (accession number GCF_000001735.4), with an e-value cut-off of 1e − 3 [32–34]. *C. baccatum* and *Capsicum chinense* were mapped to *C. baccatum* ‘PBC81’ genome assembly ASM227188v2 [3, 34]. To estimate the abundance of the annotated transcripts, we used the featureCounts tool with the default parameters [35]. We conducted the steps from mapping to quantification on the Galaxy platform [36]. All data on the expression (raw and TMM-normalized counts) and annotation of *C. annuum* ‘Dempsey’ transcripts with homologs are provided in Data S1.

### Differential expression analysis

A differentially expressed gene (DEG) analysis was conducted in two steps using edgeR and NOISeq packages [37, 38]. First, based on the Benjamini and Hochberg’s approach, the DEG analysis was performed using the edgeR package with a false discovery rate (FDR) of less than 0.05. The low-expression genes were filtered out, and the Trimmed Mean of M-values (TMM)-normalized counts were obtained based on Counts Per Million (CPM) value of more than 1 in at least one sample. Second, non-DEGs were filtered using the NOISeq package with a ranking score (RS) of 20.1, based on an Euclidean distance of the fold change (FC) of more than 2 and an absolute expression difference (D) greater than 20 between the TMM-normalized counts [38, 39]. After filtering non-DEGs, a heatmap was generated, and the subsequent DEGs were clustered into six K-means clusters using Morpheus software (https://software.broadinstitute.org/morpheus). The distance metric for clustering was the Pearson correlation coefficient. For understanding correlations across RNA-seq samples, the R function *prcomp* was used for performing the PCA analysis, the R packages *ggplot2* was used to visualize the PCA plot, and the R package *corrplot* was used to perform the Pearson’s correlation efficient analysis and visualize the correlation matrix [40–42].

### Functional enrichment analysis

To elucidate the shoot formation mechanism and gene expression in ‘Dempsey’, we annotated DEGs based on the gene ontology for specifying biological processes (GO: BP) database using the R package *org.At.tair.db* [43]. To visualize the results, a dot-plot and cnetplot were produced using the R package *clusterProfiler* v4.0, helping us understand the primary biological processes involved and identify hub genes within the K-means clusters [44].

### Comparative transcriptome analysis

For comparing the DEGs of *C. annuum* ‘Dempsey’, we selected and downloaded DEG datasets from RNA-seq data containing of ‘Seedling’, ‘Callus’, and ‘Shoot apices’ data for *P. axillaris*, *P. exserta*, and *P. integrifolia* [32], Additionally, we downloaded *A. thaliana* RNA-seq data containing CON0d [control, after-cutting < 15 mins], CIM4d [on callus-inducing media for 4 days], and SIM4d and SIM6d [on shoot-inducing media for 4 and 6 days, respectively] data, representing gene expression datasets derived from wild-type, callus, and shoot tissue [45]. To identify DEGs common among the similar tissue types of all five species (including our ‘Dempsey’ data), we used the DiVenn 2.0 program and InteractiVenn programs [46, 47]. We focused on callus and shoot-specific gene regulation by identifying DEGs between leaf/seedling and callus and leaf/seedling and shoot RNA-seq samples, thereby discerning upregulated and downregulated genes in the callus and shoot tissues compared to basal control. In the comparative datasets, we identified all DEGs using an FDR < 0.05 in ‘Dempsey’ and *A. thaliana* and a probability value (q) of > 0.8 in *Petunia* spp., using a previously described method [39]. The Arabidopsis Hormone Database 2.0 (AHD2.0) and the R package *org.At.tair.db* were utilized for analyzing the relationships between phytohormones and DEGs in the five species [43, 48].

### Chlorophyll content measurement

To confirm the deactivation of the regulatory system governing photosynthesis and chlorophyll biosynthesis, chlorophyll content was measured using a 96-well microplate and methanol extraction [49]. After measuring the fresh weight (FW) of the tissue powder, chlorophyll extraction was achieved by adding 1 mL of methanol and vortexing for 2 min. Following extraction, samples were centrifuged for two minutes at 16,760 g, and the supernatant was separated from the pellet and added to an empty 2 mL Eppendorf tube. The pellet underwent a second extraction using 1 mL of methanol and further voltexing for 2 min. After centrifuging at 16,760 g, the supernatant was transferred, the pellet was discarded, and the two supernatants were combined to measure the chlorophyll content. Chlorophyll content was calculated using Warren’s (2008) formula and normalized using the FW of the tissue powder [49].

### Quantitative real-time reverse-transcription PCR (qRT-PCR)

To validate the RNA-seq data of ‘Dempsey’, the expression profiles of genes were examined by qRT-PCR. The cDNAs were synthesized using the ReverTra Ace qPCR RT Master Mix with a gDNA Remover kit (Toyobo, Osaka, Japan). qRT-PCR was performed with the cDNA as template using PowerUp™ SYBR™ Green Master Mix (Applied Biosystems, Vilnius, Lithuania) under the following conditions: 95 °C for 10 min, followed by 40 cycles of 95 °C for 15 s and 60 °C for 1 min. All reactions were performed in three biological replicates. We utilized Actin (*CaDEM03G20100*) and GAPDH (*CaDEM03G33920*) as multiple reference genes for qRT-PCR data normalization. All primer sequences for qRT-PCR are listed in Table S1. Relative gene expression was calculated using the 2^−ΔΔCт^ method [50].

## Results

### ‘Dempsey’ transcriptomes reveal DEGs in proliferating callus tissue and emerging shoots

**Fig. 1:**
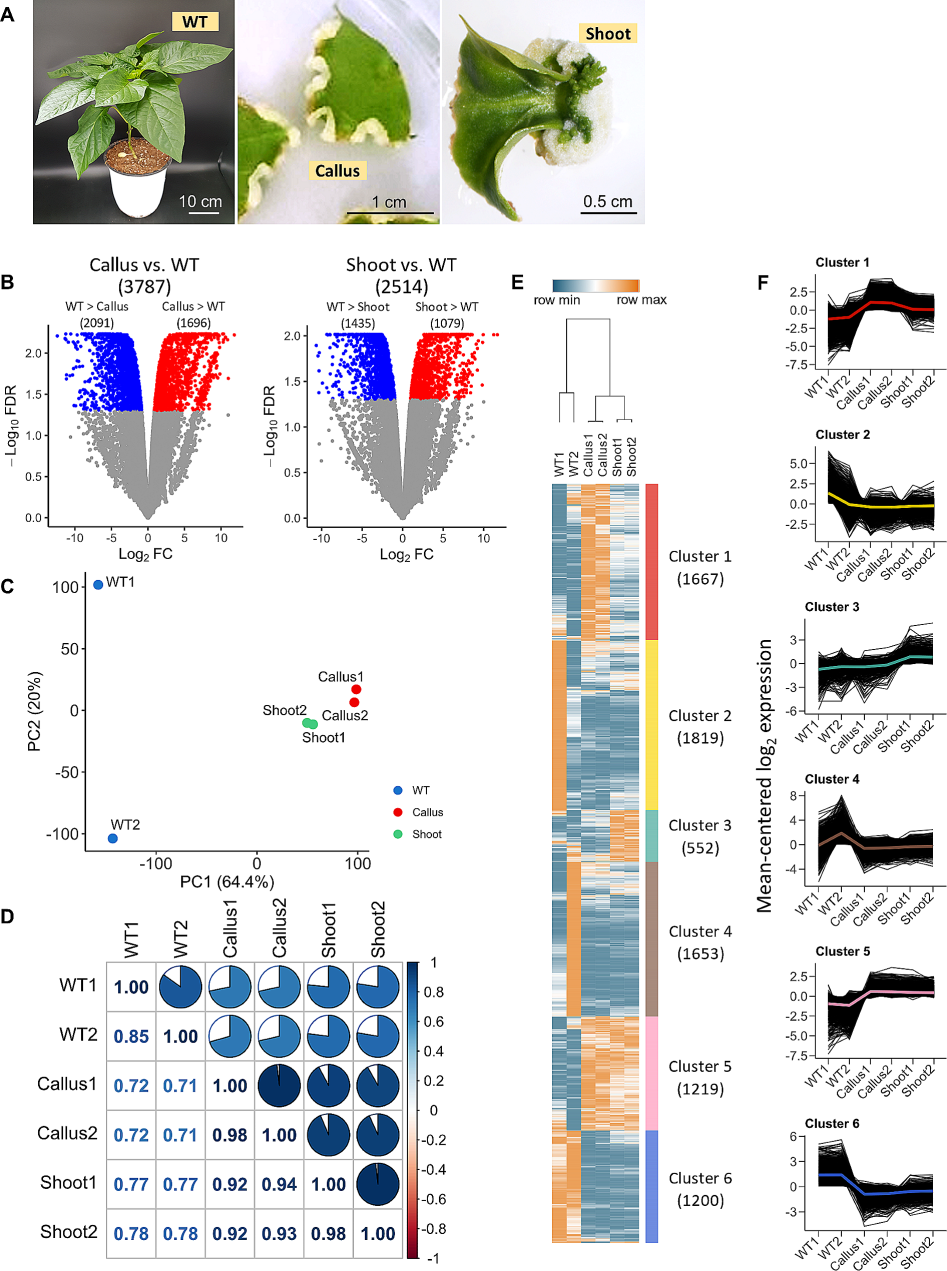
Analysis of DEGs from callus and shoot tissue in *Capsicum annuum* ‘Dempsey’. (**A**) Example images with the scale bar of samples from leaf (WT), callus, and shoot tissues used for RNA-seq analysis: ‘Dempsey’ leaf WT (left), leaf-derived callus tissue (middle), callus-derived emerging shoot tissue (right). (**B**) Volcano plots depicting the DEGs of callus versus WT (left) and shoot versus WT (right) comparisons. (**C**) Principal component analysis (PCA) plot of the TMM-normalized counts of the RNA-seq samples. (**D**) Correlation matrix plot (Corrplot) showing Pearson’s correlation efficient of RNA-seq samples. The filled fraction of the circle in each pie charts (upper) corresponds to the Pearson’s correlation coefficient (lower). Blue and red colors denote positive and negative correlations, respectively. (**E**) A heatmap of DEGs, which are grouped by K-means clustering into six clusters (colored bars) with numbers in brackets indicating the number of genes in each cluster. The X-axis represents the two biological replicates of RNA-seq samples taken from the three tissue types. The Y-axis represents individual gene expression levels, visualizing the variations in gene expression across tissue types and from the three tissue types. The Y-axis represents individual gene expression levels, visualizing the variations in gene expression across tissue types and samples. (**F**) Log_2_-transformed expression levels of genes in each K-means cluster. The X-axis represents the two biological replicates of RNA-seq samples taken from the three tissue types. The Y-axis represents the mean-centered log_2_ expression level of the genes. Each graph is marked by a line representing the mean log_2_ expression level in the color assigned to each cluster in panel E.

To explore DEGs across distinct *C. annuum* ‘Dempsey’ tissues, we collected total RNA sequencing results from leaf tissue (WT), leaf explant-derived callus tissue (Callus), and callus-driven emerging buds (Shoot) (Fig. 1A). BLAST results showed that 39,392 genes in the ‘Dempsey’ genome were annotated to 22,482 genes of *Capsicum annuum* ‘Zunla-1’, 22,398 genes of *Capsicum annuum* ‘UCD-10X-F1’, and 14,130 genes of *Arabidopsis thaliana*. Mapping rates of RNA-seq reads on the ‘Dempsey’ genome ranged from 98.34 to 98.61% (Table S2).

We identified 3,787 and 2,514 genes that were differentially expressed (at an FDR < 0.05) in callus tissue compared to ‘Dempsey’ leaf tissue (Callus vs. WT) and shoot tissue compared to ‘Dempsey’ leaf tissue (Shoot vs. WT), respectively. Of these, the 1,696 and 1,079 genes exhibited increased expression in Callus vs. WT and Shoot vs. WT, respectively. In contrast, 2,091 and 1,435 genes exhibited decreased expression in Callus vs. WT and Shoot vs. WT, respectively (Fig. 1B).

The transcriptomic data were simplified using a PCA analysis to better understand the relationships among the tissue type samples. As a result, the first and second principal components (PC1 and PC2) explained 64.4% and 20.0% of the total variation, respectively (Fig. 1C). Through a pairwise comparison of the RNA-seq samples, a notable difference was observed between the WT libraries and the libraries from the Callus and Shoot, most conspicuously in the PC1 (Fig. 1C). According to 20.0% of the PC2, the Pearson’s correlation coefficient of each tissue group was 85.0 in the WT and 0.98 in the Callus and Shoot, thereby representing high sample similarities in the positive correlation (Fig. 1C, D).

By applying the criteria of CPM greater than 1 in edgeR and an RS greater than 20.1 in NOISeq, excluding low-expression genes with non-expression patterns, 8,110 cluster signature genes were obtained. After filtering featureless genes, we identified 6 K-means clusters containing 552 genes in the smallest cluster and 1,819 in the largest (Fig. 1E). Cluster 1, containing 1,667 genes, predominantly showed upregulated genes associated with callus tissue; Cluster 3, containing 552 genes, predominantly showed upregulated genes associated with shoot tissue; and Cluster 5, containing 1,219 genes, showed upregulated genes associated with both callus and shoot tissues. These clusters were distinct from clusters 2, 4, and 6, which contained 1,819, 1,653, and 1,200 genes, respectively (Fig. 1E). These Clusters showed high expression levels of WT samples. Specifically, Cluster 2 and 4 displayed unique gene expression patterns for WT1 and WT2, possibly reflecting distinct biological variations. In contrast, Cluster 6 exhibited gene expression common to both WT samples, indicating a leaf-specific expression profile (Fig. 1E, F).

### Gene ontology (GO) enrichment analysis reveals genetic features of callus and shoot formation

**Fig. 2:**
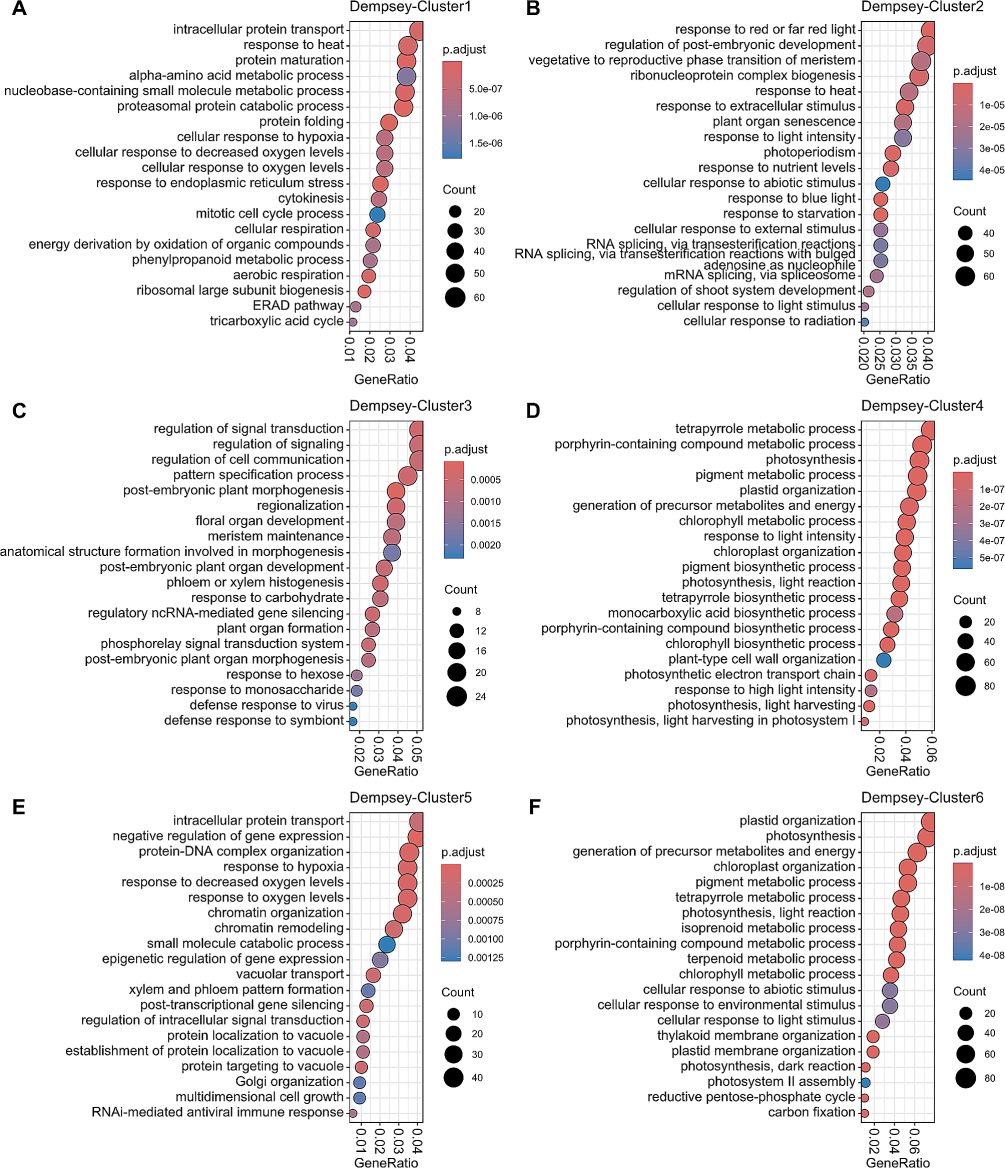
Visualization of GO terms (Y-axis) representing biological processes for K-means clusters in *Capsicum annuum* ‘Dempsey’. (**A**) Cluster 1; (**B**) Cluster 2; (**C**) Cluster 3; (**D**) Cluster 4; (**E**) Cluster 5; (**F**) Cluster 6. The dot color represents the adjusted p-value (p.adjust; −log_10_[FDR]). The dot size represents the number of DEGs representing each GO term (Count). The X-axis indicates the number of DEGs in each GO term relative to the total number of genes in each K-means cluster (GeneRatio)

In the GO enrichment profile of callus-specific Cluster 1, cell division was highlighted during callus proliferation by the ‘cytokinesis’ and ‘mitotic cell cycle process’ annotations, and mitochondrial energy processes were exhibited in the ‘cellular respiration’, ‘energy derivation by oxidation of organic compounds’, and ‘tricarboxylic acid cycle’ annotations (Fig. 2A).

The WT-specific clusters 2, 4, and 6 were enriched with genes involved in photosynthetic processes and chloroplast development, with annotations such as ‘photosynthesis’, ‘chloroplast organization’, ‘pigment metabolic process’, ‘response to light intensity’, ‘tetrapyrrole metabolic process’, ‘photosynthetic electron transport chain’, ‘reductive pentose-phosphate cycle’, and ‘carbon fixation’ (Fig. 2B, D, F).

Shoot-specific Cluster 3 was particularly enriched with genes implicated in the phytohormone-activated signaling pathway and cell differentiation during shoot formation, including annotations in ‘regulation of signal transduction’, ‘regulation of cell communication’, ‘pattern specification process’, ‘post-embryonic plant morphogenesis’, ‘regionalization’, ‘meristem maintenance’, ‘anatomical structure formation involved in morphogenesis’, ‘phloem or xylem histogenesis’, and ‘plant organ formation’ (Fig. 2C). Additionally, Cluster 3 was enriched with genes involved in the response to monosaccharides as a metabolic feature and genes responsive to pathogens, such as those in the defense responses to viruses and symbionts as biotic stressors (Fig. 2C).

Cluster 5, which comprised genes with high expression in both callus and shoot tissues, included gene annotations involved in the immune system, such as ‘RNAi-mediated antiviral immune response’; developmental processes, such as ‘xylem and phloem pattern formation’ and ‘multidimensional cell growth’ (Fig. 2E). Clusters 1 and 5 were also enriched in genes involving the protein modification and recycling for callus formation, with annotations such as ‘intracellular protein transport’, ‘protein maturation’, ‘alpha-amino acid metabolic process’, ‘proteasomal protein catabolic process’, ‘protein folding’, ‘ERAD pathway’, and ‘response to hypoxia’ (Fig. 2A, E).

### Defense mechanisms and hypoxia responses are involved in callus growth and shoot formation

In clusters 1, 3, and 5, genes associated with defense mechanisms and hypoxia showed high expression levels based on the GO enrichment analysis. Thus, we investigated DEGs involved in defense and hypoxia within these clusters. Multiple defensins (*CaDEM07G00190*, *CaDEM07G00200*, *CaDEM07G01550*, *CaDEM07G01560*, and *CaDEM12G07160*) in clusters 1 and 5 were highly upregulated (log_2_ fold changes [log_2_|FC|] > 5.9) in ‘Dempsey’ callus tissue (Table S3). Lignin-based pathogen barrier-forming CASP-like proteins (*CaDEM05G03350* and *CaDEM05G03420*) in clusters 1 and 5 were also highly upregulated (log_2_|FC| > 5.5) (Table S3). Moreover, we found 21, 4, and 5 upregulated peroxidases belonging to clusters 1, 3, and 5, respectively (Table S3). Thus, we revealed a potential link indicating that the hypoxic condition in callus tissue was due to the limitation of oxygen diffusion by respiratory bursts or lignin barriers, each acting as a defense mechanism during callus and shoot formation (Table 1). This is examined in more depth in the Discussion Sect. 4.2.

**Table 1:** Characterization of the gene groups involved in defense and hypoxia responses

Reference	Characterization
Defensins	
Stotz et al. (2009)	Biotic and abiotic stimuli induce the expression of the defensins: (1) environmental stress, such as drought, salt, and cold, and (2) phytohormones, such as ET, JA, and SA. Certain plant defensins exhibit inhibiting proteinases and α-amylases and obstructing protein translation, which may enhance their effectiveness in plant defense mechanisms.
Nickel et al. (2012)	Hypoxia triggers the upregulation of the vitamin D receptor and its downstream target, the antimicrobial human β defensin 2 (hBD2)
Khan et al. (2019)	Plant defensins possess robust defense mechanisms against fungal pathogens and responsiveness to abiotic stresses. Defensins accelerate ROS production.
CASP-like proteins	
Lee et al. (2019)	Casparian strip membrane domain protein (CASP)-like proteins are required for pathogen-induced lignification by spatial restriction of pathogens. The lignin polymerization is involved in ROS production regulated by NADPH oxidases or respiratory burst oxidase homologs (RBOHs).
Peroxidases	
Naseer et al. (2012)	Localized Casparian strip (CS) formation is facilitated by restricting the activity of lignin polymerization to a ring-shaped zone around the cell’s meridian. This restriction can be accomplished by targeting lignin-polymerizing enzymes like peroxidases to the specific area. Additionally, it can involve the confinement of ROS production to this region or the directed movement of monolignol substrates to the same localized area.
Lee et al. (2019)	Specific peroxidases play a crucial role in CS formation by lignin polymerization in root endodermal cells. The CS acts as a diffusion barrier, effectively blocking the movement of water and solutes within the root endodermis.
Xiao et al. (2022)	Lignin peroxidase of phytopathogenic fungi (*Botryosphaeria kuwatsukai*) behaves as a microbe-associated molecular pattern (MAMP) to trigger the defense response of plants, including cell death, ROS burst, callose deposition, and upregulation of immunity-related genes.

### Gene concept network analysis illustrates key hub genes for callus growth and shoot formation

**Fig. 3:**
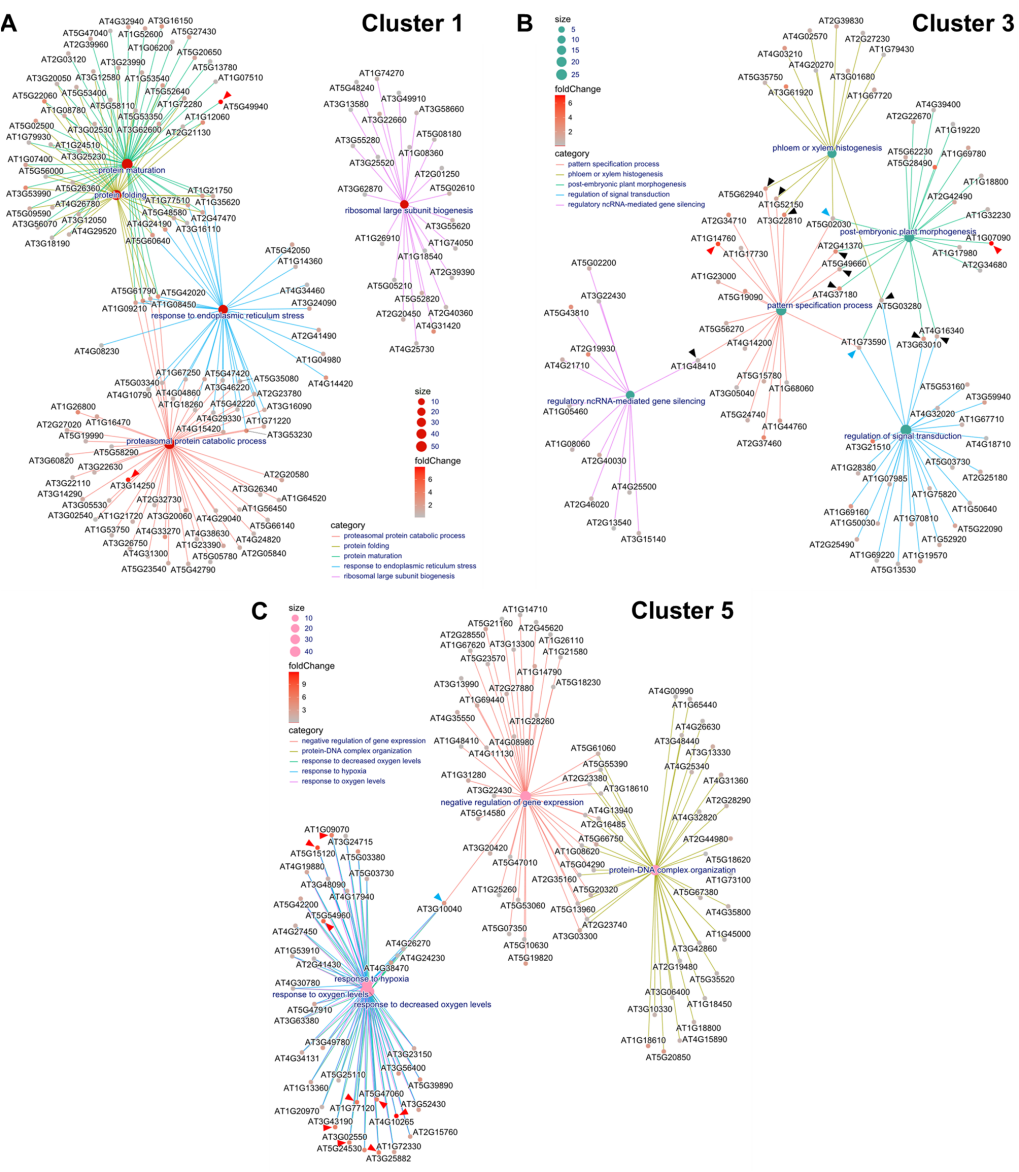
Gene-concept network (Cnetplot) depicting gene-to-GO term relationships in *Capsicum annuum* ‘Dempsey’. (**A**) the callus-specific cluster (Cluster 1); (**B**) the shoot-specific cluster (Cluster 3); (**C**) the cluster representing both callus and shoot tissue DEGs (Cluster 5). The cnetplots visualize the top 5 significantly enriched GO terms and the genes related to those GO terms in each cluster (category). The size of dots at the center of each cluster represents the number of genes related to the associated GO term (size). The vertical color bar indicates the log_2_|fold change| in gene expression for each gene (foldChange). Red arrowheads indicate an extreme change in gene expression (log_2_|fold change| > 5). Blue arrowheads indicate key ternary or quaternion hub genes providing high connectivity among the morphogenesis or hypoxia-related GO terms. Black arrowheads indicate key binary hub genes providing high connectivity among the morphogenesis or hypoxia-related GO terms

Using gene-concept network plots (cnetplots), we visually portrayed the intricate gene networks of clusters 1, 3, and 5, showcasing the interaction among DEGs and the top five significantly enriched GO terms pertinent to callus and shoot formation in *C. annuum* ‘Dempsey’ (Fig. 3). The cnetplots also exhibit ‘hub genes’, i.e., those having a high level of connectivity within the gene network among the top five significantly enriched GO terms (Fig. 3). Hub genes could be highly influential in each cluster because they may regulate or be regulated by many other genes, suggesting their importance for understanding critical regulatory mechanisms in biological processes. The interconnected representation in the plots provide a concise overview of the associations between genes and GO terms in callus-specific Cluster 1 (Fig. 3A), shoot-specific Cluster 3 (Fig. 3B), and callus/shoot-specific Cluster 5 (Fig. 3C).

In callus-specific Cluster 1, the significant increase in major biological processes indicated 27 hub genes centered around the GO term ‘response to endoplasmic reticulum (ER) stress’, which shared co-expression in the primary biological processes of ‘protein maturation’, ‘protein folding’, and ‘proteasomal protein catabolic process’ (Fig. 3A). In Cluster 1, transcription levels of the *AT5G49940* homologue, the iron-sulfur cluster assembler *NFU2* (*CaDEM01G12270*), and *AT3G14250* homologues, *RBR E3 ubiquitin ligase* genes (*CaDEM03G39660* and *CaDEM03G41470*), were significantly increased, with log_2_|FC| values of 7.9, 7.6, and 5.9, respectively, in the Callus vs. WT comparison. Co-expression of the three genes in ‘Dempsey’ was shared by the response to ER stress (Fig. 3A, Data S1).

In shoot-specific Cluster 3, prominent gene expression was observed for *AT1G14760* homologue, *KNATM* (*CaDEM06G26780*), and *AT1G07090* homologue, *LSH6* (*CaDEM05G03950*), with expression log_2_|FC| values of 5.8 and 7.1, respectively, in the Shoot vs. WT comparison (Fig. 3B). Of the top five GO terms, two key hub genes were observed within the ternary GO network. The *AT5G02030* homologue, BEL1-like homeodomain *RPL* (*CaDEM09G08050*), was the hub among GO terms ‘phloem or xylem histogenesis’, ‘post-embryonic plant morphogenesis’, and ‘pattern specification process’. The *AT1G73590* homologue, auxin efflux carrier *PIN1* (*CaDEM03G42500*), was a hub gene among ‘phloem or xylem histogenesis’, ‘post-embryonic plant morphogenesis’, and ‘regulation of signal transduction’. The roles of these two hub genes, *RPL* and *PIN1*, in Cluster 3, indicates their involvement shoot differentiation and growth in the ‘Dempsey’ cultivar. In particular, ‘pattern specification process’ and ‘post-embryonic plant morphogenesis’ GO terms are known to be associated with shoot growth (Fig. 3B).

Another ten hub genes were discovered within binary GO networks in Cluster 3 (Fig. 3B). As morphogenesis-related gene expression, ‘phloem or xylem histogenesis’- and ‘pattern specification process’-associated genes were connected with three hub genes: *AT5G62940* homologue, *HCA2* (*CaDEM03G36060*); *AT1G52150* homologues, *ATHB-15* (*CaDEM12G18880* and *CaDEM03G44680*); and *AT3G22810* homologue, *FL2* (*CaDEM03G14860*). GO terms ‘pattern specification process’ and ‘post-embryonic plant morphogenesis’ were connected via the *AT2G41370* homologue, *BOP2* (*CaDEM05G00340*); *AT5G49660* homologue, *XIP1* (*CaDEM04G21220*); and *AT4G37180* homologue, *UIF1* (*CaDEM02G29970*). ‘Phloem or xylem histogenesis’ and ‘regulation of signal transduction’ were linked by the *AT5G03280* homologue, *EIN2* (*CaDEM09G02640*), an ethylene signal transduction-related gene. ‘Post-embryonic plant morphogenesis’ and ‘regulation of signal transduction’ were connected by two hub genes, *AT3G63010* homologue, *GID1B* (*CaDEM06G00810*), and *AT4G16340* homologue, *SPK1* (*CaDEM01G40660*). Lastly, ‘regulatory ncRNA-mediated gene silencing’- and ‘pattern specification process’-annotated genes were linked through the *AT1G48410* homologue, *AGO1* (*CaDEM06G26600*).

In Cluster 5, sharing upregulation of both callus and shoot, three hypoxia-related GO terms were noticeably selected as top five GO terms in Cluster 5, including ‘response to oxygen levels’, ‘response to decreased oxygen levels’, and ‘response to hypoxia’ (Fig. 3C). The expression of nine genes was significantly increased in all three of these terms (log_2_|FC| > 5.0) in the Callus vs. WT comparison: *AT1G09070* homologue, *SRC2* (*CaDEM08G01510*), *AT5G15120* homologue, *PCO1* (*CaDEM03G36330*), *AT5G54960* homologue, *PDC2* (*CaDEM02G17450*), *AT1G77120* homologue, *ADH1* (*CaDEM04G14000*), *AT5G47060* homologue, *DUF581* (*CaDEM06G16370*), *AT4G10265* homologue, *WIP3* (*CaDEM07G20060*), *AT3G43190* homologue, *SUS4* (*CaDEM09G25130*), *AT3G02550* homologue, *LBD41* (*CaDEM03G43310*), and *AT3G25882* homologue, *NIMIN-2* (*CaDEM03G43370*) (Fig. 3C). The hypoxia response attenuator 1 (*HRA1*) homologue, *AT3G10040* (*CaDEM09G01260*), was a quaternion hub gene mediating ‘response to hypoxia’, ‘response to oxygen levels’, ‘response to decreased oxygen levels’, and ‘negative regulation of gene expression’ (Fig. 3C). Thus, our analysis revealed that hypoxia may strongly influence the gene expression patterns in Cluster 5, which was associated with both callus and shoot tissues.

### Comparative transcriptomic analyses of five species identified the conserved essential genes for callus and shoot development

To get mainly conserved genetic features for shoot formation in *C. annuum* ‘Dempsey’, we conducted a comparative analysis using DEG datasets derived from the RNA-seq data of *Petunia axillaris*, *Petunia exserta*, *Petunia integrifolia*, and *A. thaliana.*

**Fig. 4:**
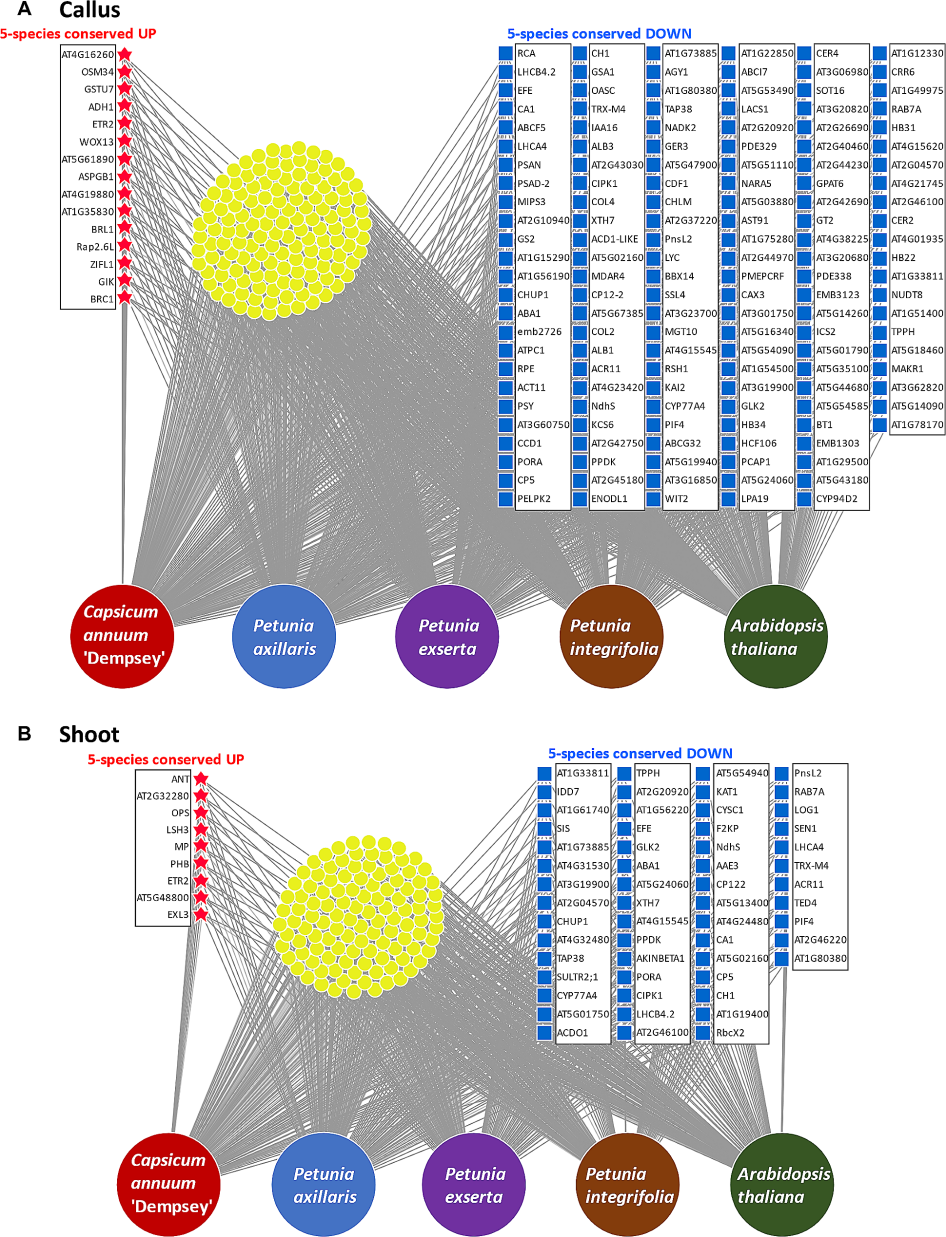
DiVenn diagrams depicting the conserved gene regulation patterns of five species: *Capsicum annuum* ‘Dempsey’, *Petunia axillaris*, *Petunia exserta*, *Petunia integrifolia*, and *Arabidopsis thaliana.* (**A**) callus tissues of five species; (**B**) shoot tissues of five species. Red stars indicate the upregulated genes common among the five species (5-species conserved UP), while blue squares denote the common downregulated genes (5-species conserved DOWN)

This analysis revealed that 15 genes exhibited increased expression in callus tissue when compared to the basal control across all five species (Fig. 4A, Fig. S1A). Based on GO term profiling, three of these genes were involved in defense mechanisms (*AT4G16260*, *OSM34*, and *AT5G61890*), three with hypoxia (*ADH1*, *ETR2*, and *AT4G19880*), and one each with the development of callus (*WOX13*) and shoot (*BRC1*) tissues (Fig. 4A, Data S2). Meanwhile, 146 genes showed decreased expression in all species’ callus tissue. These were involved in broad and varied biological functions (Fig. 4A, Fig. S1B, Data S2): ten genes were related to photosynthesis (*LHCB4.2*, *CA1*, *LHCA4*, *PSAN*, *PSAD-2*, *PORA*, *ALB1*, *PPDK*, *RSH1*, and *NARA5*), ten to chloroplast organization and movement (*AT1G15290*, *CHUP1*, *ALB3*, *AT5G67385*, *COL2*, *CDF1*, *GLK2*, *HCF106*, *EMB3123*, and *EMB1303*), and seven to chlorophyll biosynthesis (*PORA*, *CH1*, *GSA1*, *ALB1*, *CHLM*, *GLK2*, and *EMB1303*) (Fig. 4A). These three functions implicate a reduction in photosynthetic activity in calli across all five species.

Additionally, nine genes exhibited increased expression in shoot tissues compared to the basal control across all five species (Fig. 4B, Fig. S1C). Of these, six were involved in cell differentiation and development (*ANT*, *AT2G32280*, *OPS*, *LSH3*, *MP*, and *PHB*) and one each were involved in hypoxia response (*ETR2*) and protein ubiquitination (*AT5G48800*), and one had an unknown function (*EXL3*) (Fig. 4B, Data S3). Among these genes, *ANT* plays a role in the primordial outgrowth of the shoot, *LSH3* suppresses cell division in shoot organ boundaries, *VCC* (*AT2G32280*) and *OPS* play roles in the development of early provasculature development through auxin maxima, and *ETR2* inhibits root elongation through ET. At the same time, 56 genes showed decreased expression in the shoots of all species (Fig. 4B, Fig. S1D, Data S3). These were involved in various biological functions, including photosynthesis (*TAP38*, *ACDO1*, *PPDK*, *PORA*, *LHCB4.2*, *NdhS*, *CA1*, *RbcX2*, *PnsL2*, *LHCA4*, *TED4*, and *AT1G80380*), chloroplast organization and movement (*GLK2* and *CHUP1*), chlorophyll biosynthesis (*ACDO1*, *GLK2*, *PORA*, and *CH1*), fatty acid metabolism (*CYP77A4*, *EFE*, and *AKINBETA1*), cell wall biogenesis (*XTH7*), ER body organization (*AT4G15545*), photomorphogenesis and skotomorphogenesis (*PORA*), hypoxia response (*AT5G54940*), defense mechanisms and stress responses (*EFE*, *AAE3*, and *CA1*), and the regulation of monosaccharides or decreased response to disaccharides (*TPPH*, *F2KP*, and *ACR11*) (Fig. 4B).

Interestingly, *ETR2*, a gene associated with responses to hypoxia and ET, was the sole gene upregulated in both callus and shoot tissue (Fig. S1E, Data S2 and S3), while decreased expression of genes associated with photosynthetic regulation (*TRX-M4*, *GLK2*, and *PIF4*), photosynthesis (*LHCB4.2*, *LHCA4*, *NdhS*, *PnsL2*, *PORA*, *CH1*, *CA1*, and *PPDK*), chloroplast movement (*CHUP1*), response to sucrose (*ACR11*), and trehalose biosynthesis (*TPPH*) were shown in both callus and shoot tissue (Fig. S1F, Data S2, and Data S3). The total chlorophyll a and b content of the Callus (average in 0.14 mg/g FW) and Shoot (average in 0.22 mg/g FW) was significantly decreased compared to the WT (average in 0.58 mg/g FW), supporting a reduction in photosynthetic activity and chlorophyll biosynthesis in the callus and shoot tissues (Fig. S2). Therefore, we discovered an overall decline in photosynthesis-related gene expression in regenerative tissues, indicating a shut-down of the regulatory mechanism for photosynthesis.

### A high proportion of auxin-related genes are conserved in ‘Dempsey’ for *de novo* shoot formation

**Fig. 5:**
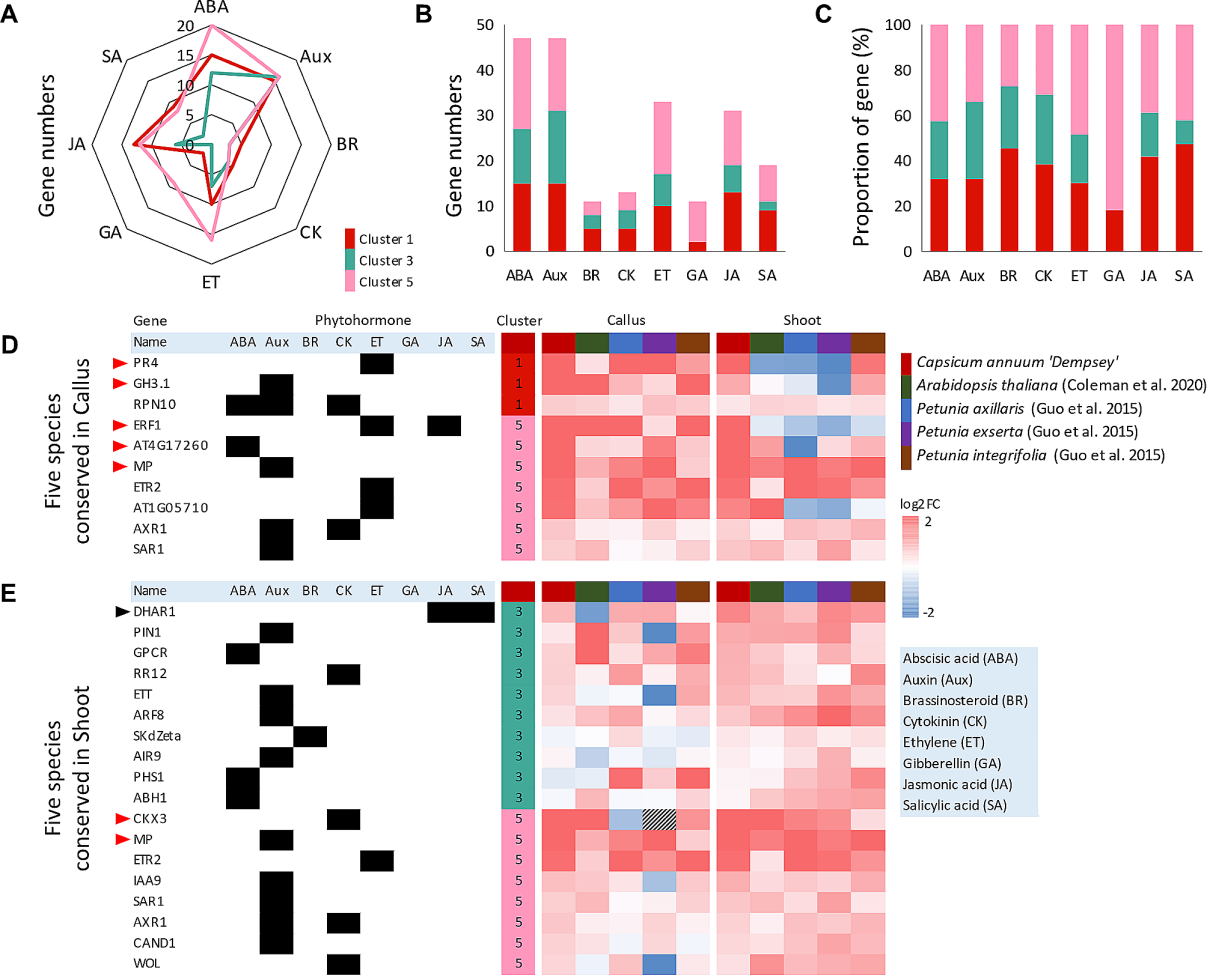
Phytohormone-associated genes belonging to callus-specific Cluster 1 (red), shoot-specific Cluster 3 (aqua), and the cluster representing both callus and shoot tissue, Cluster 5 (pink), in *C. annuum* ‘Dempsey’. (**A**) a polar plot of phytohormone-related genes in each K-means cluster, with the eight phytohormones represented by each pole (see the light blue box for phytohormone abbreviations); (**B**) a stacked bar plot showing the gene numbers in each K-means cluster (colors) for each phytohormone (X-axis); (**C**) a proportional stacked bar plot of the genes in each K-means cluster (colors) for each phytohormone (X-axis); (**D**) heatmap of comparing the transcriptomes of the five species in callus tissue; (**E**) heatmap of comparing the transcriptomes of the five species in callus tissue. RNA-seq data were analyzed to identify phytohormone-related DEGs in each cluster with expression of *C. annuum* ‘Dempsey’ (red), *A. thaliana* (green), *P. axillaris* (blue), *P. exserta* (purple), and *P. integrifolia* (brown). The color scale bar of heat intensity indicates the log_2_-transformed fold change (log_2_|FC|) in expression (the grey box on the heatmap indicates no recorded expression). Red arrowheads indicate highly upregulated genes (log_2_|FC| > 2). The black arrowhead indicates the most upregulated gene (a log_2_|FC| of 1.5–2) for shoot formation (Cluster 3). The black boxes to the left of the heatmaps indicate the phytohormone(s) related to each gene

To further investigate the significant genes influencing shoot formation in the ‘Dempsey’ cultivar, we used a comparative transcriptome analysis focusing on with phytohormone-related genes, categorizing clusters 1, 3, and 5 according to their prevalent phytohormonal relationships (Fig. 5A, B, C). The three clusters revealed distinct relationships with eight phytohormones: ABA, auxin, cytokinin (CK), ET, gibberellic acid (GA), BR, JA, and salicylic acid (SA) (Fig. 5A, B, C). Callus-specific Cluster 1 showed a notable linkage with, in descending order, the hormones ABA, auxin, JA, ET, and SA (15, 15, 13, 10, and 9 genes, respectively) (Fig. 5A, B). Shoot-specific Cluster 3 was mainly related to auxin and ABA (16 and 12 genes, respectively) (Fig. 5A, B). Cluster 5 was highly related to ABA, auxin, ET, JA, and GA (20, 16, 16, 12, and 9 genes, respectively) (Fig. 5A, B). Clusters 1 and 5 were at least marginally associated with all eight phytohormones (Fig. 5A, B), indicating that they all played differential roles in the gene expression of comparative tissues of ‘Dempsey’. In the context of *de novo* regeneration, focusing on the phytohormone-related genes of clusters 1, 3, and 5, the proportion of genes in Cluster 1 associated with each phytohormone category were SA (47.4%), BR (45.5%), JA (41.9%), CK (38.5%), ABA (31.9%), auxin (31.9%), ET (30.3%), and GA (18.2%) (Fig. 5C). Meanwhile, the proportion of genes in shoot-forming Cluster 3 for each phytohormone category were auxin (34.0%), CK (30.8%), BR (27.3%), ABA (25.5%), ET (21.2%), and JA (19.4%), while SA was less strongly associated (10.5%) and GA was not involved with Cluster 3 genes (Fig. 5C).

To better understand the primarily phytohormone-related genes involved in shoot formation, we investigated conserved phytohormone-related genes in five species. This showed that phytohormonal gene regulation varied in the callus and shoot transcriptomes of the five species (Fig. 5D, E). In the comparative callus transcriptomes (Fig. 5D), the grouping of clusters 1 and 5 was interpreted as relating to regulation of callus formation before shoot development (Cluster 3). In the comparative shoot transcriptomes (Fig. 5E), the grouping of clusters 3 and 5 was interpreted as regulation of shoot formation occurring after or during and after callus development, respectively.

For the calli of the five species, we revealed phytohormone-related gene regulations of three genes that were conserved among the five species in Cluster 1 and seven in Cluster 5 (Fig. 5D). Of these, *PR4* and *GH3.1* of Cluster 1 and *ERF1*, *AT4G17260*, and *MP* of Cluster 5 showed prominent upregulation (log_2_|FC| > 2) in callus tissue (Fig. 5D, Data S4). For the shoots of the five species, we found ten genes in Cluster 3 and eight in Cluster 5. Of these, *DHAR1* was the most upregulated in Cluster 3 (log_2_|FC| = 1.6) (Fig. 5E, Data S4) and *CKX3* and *MP* showed prominent upregulation in Cluster 5 (log_2_|FC| > 2) during the shoot formation (Fig. 5E, Data S4). Thus, the *MP* of Cluster 5 was prominently upregulated for overall callus and shoot development (Fig. 5D, E). Multispecies phytohormone-related gene regulation showed that auxin-associated genes represented the highest proportion of genes in both callus (50%) and shoot (50%) tissues (Fig. 5D, E). Therefore, these results indicated that in all five species, including *C. annuum* ‘Dempsey’, *de novo* callus and shoot formation was primarily related to auxin (Fig. 5D, E).

To validate the accuracy of the RNA-seq transcriptomic analyses, we performed qRT-PCR for five DEGs in shoot-specific genes (*ANT*, *MP*, *PIN1*, *LSH3*, and *PHB*), based on our comparative transcriptome results. The qRT-PCR results corroborated the RNA-seq data, demonstrating a significant increase in the mRNA levels of all five DEGs in Shoot vs. WT (Fig. S3). Among these genes, *MP*, *LSH3*, and *PHB* showed considerable shifts in gene expression levels, observed in both Callus vs. WT and Shoot vs. WT (Fig. S3), reinforcing the pivotal functions that these genes play in mediating cellular differentiation processes for *de novo* shoot formation. This concordance between the qRT-PCR and RNA-seq data not only confirmed the reliability of our transcriptomic analyses but also underscored the critical role of auxin signaling pathways in *de novo* shoot development of plants.

## Discussion

### Allocation of energy resources during callus formation in ‘Dempsey’

A significant suppression of photosynthesis was found in the pluripotent callus of rice [51]. Similarly, a loss of chlorophyll was also observed in ‘Dempsey’ callus tissue (Fig. 1A, S2). The transcriptomic analysis showed that downregulated genes in both callus and shoot tissue outnumbered upregulated genes (Fig. 1B), signifying many negatively regulated pathways in reproductive tissues compared to leaf tissue (WT). Furthermore, the DEGs found in reproductive tissues and those in leaf tissue showed incompatible gene expression patterns, and their representative biological processes, *de novo* shoot formation and photosynthesis, appeared to have an antagonistic relationship as a balanced mechanism in ‘Dempsey’ (Fig. 1E, F, and 2).

The WT-specific clusters, clusters 2, 4, and 6, were enriched in genes involved in photosynthetic processes and chloroplast development, represented by GO terms such as ‘photosynthesis’, ‘chloroplast organization’, ‘pigment metabolic process’, ‘generation of precursor metabolites and energy’, ‘response to light intensity’, ‘tetrapyrrole metabolic process’, ‘photosynthetic electron transport chain’, ‘reductive pentose-phosphate cycle’, and ‘carbon fixation’ (Fig. 2B, D, F). These biological processes are common in plant gene expression analyses, being important for the coordination of photosynthetic activity, light response, and the metabolic processes needed to support energy production and growth from light [52]. Therefore, the photosynthetic processes represented in clusters 2, 4, and 6 were incompatible with the callus- and shoot-specific processes in clusters 1, 3, and 5 (Fig. 2).

As the common pattern seen in the three *Petunia* spp., Arabidopsis, and ‘Dempsey’, the simultaneous increase in the expression of genes for callus and shoot development and decrease in the expression of photosynthetic-related genes could indicate a strategic trade-off made by the plant (Fig. 4A, B, and S1). For instance, in situations of limited light availability, a plant might focus more on growth to reach light via skotomorphogenesis, reducing de-etiolation and maintaining high levels of photosynthesis [53]. Thylakoid modulation to regulate photosynthesis via etiolation/de-etiolation could also reflect a particular developmental stage where the plant prioritizes rapid shoot growth or a stress response leading to the redistribution of the plant’s resources based on environmental cues or challenges [54]. Thus, we consider the allocation of energy sources in plants may prioritize callus and shoot formation over energy production through photosynthesis. Reduced activities of photosynthetic genes lead to diminished adenosine triphosphate (ATP) production in the regenerative calli [55].

Recently, adenosine monophosphate (AMP), an oxidized form of ATP, has been identified as an enhancer for shoot formation on pluripotent calli [56]. Herein, the callus-specific expression of ‘Dempsey’, Cluster 1 included a large number of ‘nucleobase-containing small molecule metabolic process’-related genes, indicating that this was a noteworthy biological activity during callus formation (Fig. 2A). Hence, molecules involved in energy metabolism are thought to play a crucial role in promoting shoot formation; however, the detailed molecular mechanisms remain unknown due to inconsistent findings related to cyclic adenosine monophosphate (cAMP), adenosine diphosphate (ADP), and ATP [56]. Nevertheless, AMP might be essential for *de novo* shoot formation from callus tissue in ‘Dempsey’. Previous transcriptomic results from AMP-treated and CK-treated plants displayed minimal overlap, suggesting AMP is a crucial metabolite for regenerating competence in calli during tissue culture conditions associated with hypoxia [56].

### New insights into the effects of hypoxia and oxidative stress on *de novo* callus and shoot formation

The transcriptome analyses of shoot development in *C. annuum* ‘Dempsey’ highlighted overall roles of stress responses. GO enrichment analyses included diverse reactions to environmental stressors, such as wounding and hypoxia, and immune activities related to pathogens and symbiont responses, all of which contribute to the plant’s overall stress responses (Fig. 2A, C, E). Moreover, the multi-species comparative transcriptome analysis indicated molecular markers of defense mechanism (*PR4*, *GH3.1*, and *ERF1*) and hypoxia (*ADH1*, *ETR2*, and *AT4G19880*) across all five species during callus formation (Figs. 4A and 5D).

In the context of defense mechanisms, *PR4*, a pathogenesis-related gene, functions primarily in plant defense by contributing to local acquired resistance against necrotrophic pathogens, typically through its involvement in the JA signaling pathway [57]. The gene *GH3.1*, an IAA-amido synthetase, regulates hormonal balance by conjugating hormones to amino acids, affecting both plant growth and defense responses [58]. The gene *ERF1* integrates JA and ET signals in plants to activate defense genes against pathogens and herbivores [59]. Thus, responses to biotic stressors mediated by JA, auxin, and ET in callus tissue was conserved across all five species.

In hypoxia, cell damage creating wound tissue may induce a hypoxic condition because increased respiration by defense responses may result in oxygen depletion [60, 61]. Additionally, dense tissues lacking intercellular air spaces—such as in phloem and bulky, lignin-containing organs like seeds and fruits—may restrict oxygen flow, causing hypoxia [60]. Indeed, lignin polymerized by CASP-like proteins and peroxidases can block water and solute movement [62, 63]. Leaves accumulate lignin in response to bacterial pathogens through CASP-like proteins, forming a physical barrier similar to the Casparian strip in roots, thereby restricting pathogen spread and inhibiting their growth [62]. When lignin nanoparticles are tightly embedded in an artificial cellulose fiber membrane, the material properties show reduced oxygen permeability through the membrane [64]. Clusters 1, 3, and 5 of the ‘Dempsey’ transcriptome included genes associated with hypoxia response and defense mechanism-related lignin polymerization, such as those coding defensins, CASP-like proteins, and lignin-forming peroxidases (Table 1, Table S3). Furthermore, fungal lignin peroxidases can trigger the defense response of plants, including cell death, reactive oxygen species (ROS) bursts, callose deposition, and the upregulation of immunity-related genes [65]. Given these results, we consider that hypoxia in the callus tissue of ‘Dempsey’ was possibly caused by lignin barriers derived from defense responses.

Under hypoxic conditions, mitochondria become a major source of ROS, partly due to the partial reduction of oxygen, leading to the formation of superoxide anions and hydrogen peroxide (Nathan & Cunningham-Bussel 2014). Moreover, lignin polymerization confines ROS production to specific regions [63]. Our GO enrichment results based on the ‘Dempsey’ transcriptome showed cell division activities and cytokinesis in Cluster 1 genes and multidimensional cell growth in Cluster 5 genes (Fig. 2A, E), while the gene expression of Cluster 3 represented morphogenesis and plant organ formation (Fig. 2E). Therefore, we also expected expression changes between clusters 1 and 5, where the expression patterns are thought to be underlying growth and development processes triggered by hypoxia, and Cluster 3, where the expression patterns are thought to underlie shoot formation. This is because, for escaping the depletion of oxygen and energy, the involvement of hypoxia and cell death-related genes during development plays a potential role in regulating cell survival under wound stress and submergence [60, 66–70]. A previous study by Ikeuch et al. (2022) highlighted the significant role of WUSCHEL-RELATED HOMEOBOX 13 (WOX13) in controlling tissue repair mechanisms via wound stress, in regulating WIND2 and WIND3 in callus tissue formation, with RNA-seq data pointing towards hypoxia as a key factor in this process in Arabidopsis [61]. Despite *AtWIND1* and *AtWIND3* (AT1G78080 and AT1G36060) as downregulated and upregulated DEGs, respectively, no similar differential expression patterns of WIND transcription factors were found in Dempsey and three petunia species in our transcriptome comparison (Data S1). This lack of consistent expression patterns across five species may suggest a species-specific reliance on wound and regeneration signaling pathways, underlining the complexity of plant tissue repair mechanisms.

In addition, we showed that gene expression in Cluster 3 involved responses to monosaccharides (Fig. 2C). The accumulation of monosaccharides and sucrose in plant tissues is commonly seen as a reaction to abiotic stress. Additionally, high concentrations of monosaccharides in quickly expanding young plant structures can stimulate cell proliferation and the outgrowth of new leaves [61, 71]. A convergence of stress/defense mechanism-related genes underscores the intricate balance between reproductive development and environmental adaptability [66, 72–76]. Therefore, ROS production during hypoxia may be indicative of a notable link between plant stress response and energy metabolism during callus and shoot formation in ‘Dempsey’.

The significant increases in major biological processes seen in Cluster 1 included genes centered around the GO term ‘Response to ER stress’, as seen in the cnetplot (Fig. 3A). The response to ER stress is a critical aspect of maintaining protein homeostasis in the cell [77, 78]. Upon oxidative stress due to ROS, the response to ER stress plays a vital role in the cell’s ability to manage and adapt to the accumulation of misfolded proteins in the ER [79, 80]. In Cluster 1, RBR E3 ubiquitin ligase, the *AT3G14250* homologues *CaDEM03G39660* and *CaDEM03G41470*, exhibited highly upregulated transcription (log_2_|FC| = 7.6 and 5.9) in the Callus vs. WT comparison (Fig. 3A, Data S1). These genes increase ROS and the expression of defense and antioxidant enzymes when confronted by environmental stressors [81]. Non-morphogenic calli—which, when compared to morphogenic calli—are characterized by higher hydrogen peroxide content and lower redox activity, are likely under continuous oxidative stress [51, 82]. Accordingly, redox potential against ROS and proteasomal degradation machinery may be involved in the maintenance of callus pluripotency.

### Primordia outgrowth using a polar auxin accumulation is critical for *de novo* shoot formation

Two key hub genes *KNATM* (*AT1G14760* homologue *CaDEM06G26780*) and *LSH6* (*AT1G07090* homologue *CaDEM05G03950*) found in shoot-specific Cluster 3 were strongly related to organ differentiation signals for shoot morphogenesis (Fig. 3B). The gene *KNATM* plays a role in leaf proximal–distal patterning, where it is expressed in proximal–lateral domains of organ primordia and at the boundary of mature organs [83]. The gene *LSH6* (*CaDEM05G03950* homologue), a light-responsive LSH/OBO family gene, may regulate transcription in plant organ development, particularly at the junction of the SAM and lateral organs [84]. The *PIN1* gene controls the growth direction of budding organs by directing auxin flow [14]. The BEL1-like homeodomain *RPL* (*AT5G02030* homologue *CaDEM09G08050*) interacts to regulate inflorescence growth positively, and the paralogous protein interacts positively with *STM* to regulate meristem function [85]. Ankyrin repeat and BTB/POZ domain-containing *AT2G41370* is necessary for proper leaf morphogenesis [86]. Therefore, *de novo* shoot formation in *C. annuum* ‘Dempsey’ was cooperatively regulated by the shoot-specific DEGs of Cluster 3.

Auxin is a crucial regulator in the development of vasculature, chloroplast, and meristem tissue, modulating organogenesis through interactions with biosynthesis, transport, and signaling pathways [55, 87]. Accompanied by the expression of *PIN1*, the upregulation of vascular development-related genes *VCC* and *OPS* was remarkable in the ‘Dempsey’ shoot transcriptome (Figs. 3B and 4B). Moreover, 14 genes across ‘Dempsey’, petunias, and Arabidopsis indicated that auxin was the most consistently significant phytohormone in callus and shoot development (Fig. 5D, E).

The callus-specific *WOX13* gene, generally upregulated across all five species (Fig. 4A), has previously been reported to be crucial for organ recovery following grafting, which depends on callus formation and subsequent vascular cell development mediated by auxin-responsive transcription factors [61, 87]. Thus, we consider this indicates that auxin flow is critical for vascular development by accelerating auxin maxima at shoot development locations through polar auxin transport [12–14, 88–90]. Auxin possibly leads to shoot formation as a reaction to specific environmental conditions or stresses in proliferative callus.

Comparative transcriptomic results indicated that the transcription factor *MP* seen in Cluster 5 was critical in callus and shoot development across all five species (Fig. 5D, E). Auxin-responsive MP directly triggers the transcription of the homeodomain-leucine zipper III (HD-ZIP III) family, which is crucial for specifying preprocambial cells and coordinating procambial cell identity [87]. Upregulation of *PHB*, part of the HD-ZIP III group, was also conserved across the five species. Therefore, MP regulated HD-ZIP III for *de novo* shoot formation (Fig. 4B): the expression level of *MP*, regulating the expression of auxin/cytokinin-responsive genes depending on auxin maxima, determines shoot cell fate between meristem maintenance and organ development in the central and peripheral meristem [91, 92]. Based on auxin maxima, the primordial outgrowth is stimulated by the expression of *ANT*, which induces organ formation.

**Fig. 6:**
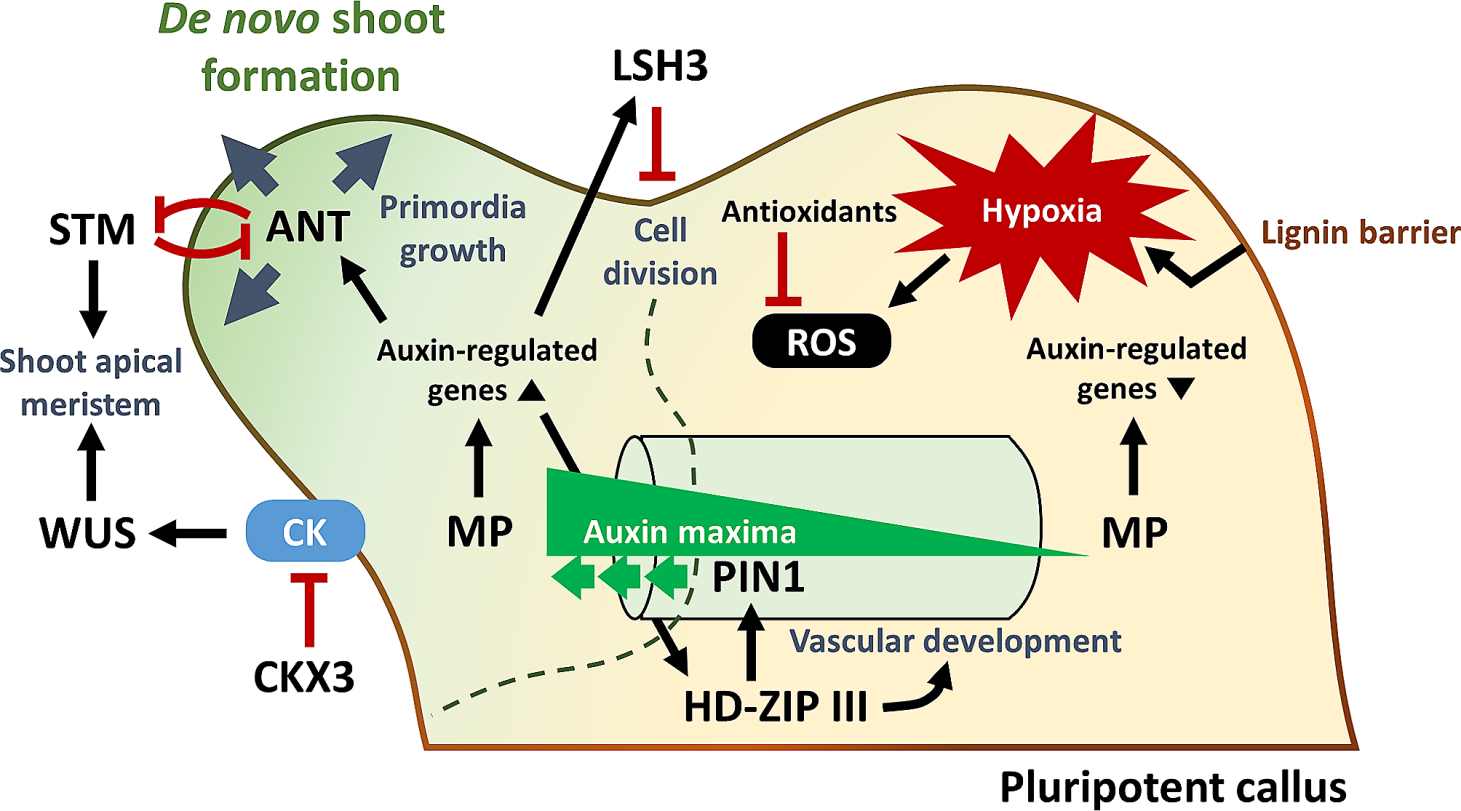
Schematic diagram of *de novo* shoot formation in *C. annuum* ‘Dempsey’ based on comparison of the transcriptomes of five species. The diagram illustrates how a hypoxic condition, caused by a low-oxygen-permeable lignin barrier, induces shoot development. This process allows for escaping oxygen and energy depletion, facilitating cell survival with ROS scavenging. For shoot morphogenesis at the escaping site, the loop of auxin-responsive regulators and the localization of auxin by the auxin efflux carrier accelerates auxin imbalance at the designated site for primordial growth and *de novo* shoot formation on the callus tissue. The antagonistic STM-CK and ANT-auxin pathways regulate the shoot apical meristem and primordia growth, respectively.At the same time, the inhibition of cell division by LSH3 establishes a boundary for the morphogenic site against the amorphous callus. Abbreviation: STM (*SHOOT MERISTEMLESS*), ANT (*AINTEGUMENTA*), LSH3 (*LIGHT SENSITIVE HYPOCOTYLS 3*), WUS (*WUSCHEL*), CKX3 (*CYTOKININ OXIDASE 3*), MP (*MONOPTEROS*), PIN1 (*PIN-FORMED1*), HD-ZIP III (class III homeodomain-leucine zipper), ROS (reactive oxygen species), CK (cytokinin)

Interestingly, the shoot transcriptome of all five species, including *C. annuum* ‘Dempsey’, indicated high expression levels of *ANT*, not *STM* (Fig. 4B, Data S1). The expression patterns of *STM* and *ANT* are mutually exclusive in the vegetative SAM, where one of these genes is active while the other is not [93]. Conserved *ANT* expression across the five species was highly upregulated, so we considered ANT-mediated primordia outgrowth to be crucial for *de novo* shoot development (Fig. 4B, Data S1). In addition, the conserved upregulation of *CKX3* across five species was found in phytohormone relationship (Fig. 5E); *CKX3* expression across all five species can be interpreted as indicating decreased cytokinin levels, which negatively affects *WUS* expression [94]. The gene *LSH3* helps to maintain the undifferentiated state of cells in tissue boundary regions during plant development, influencing organ boundary specification and meristem formation in response to auxin maxima [95]. A previous study reported that auxin positively affects callus development, suggesting its crucial role in the shoot regeneration of *C. baccatum* and *C. chinense* [3]. Our transcriptome analyses supported the finding by Shu et al. [3], particularly underscoring the significance of genes related to auxin. Moreover, we identified and confirmed the upregulation of essential genes for shoot regeneration (such as *ANT*, *MP*, *PIN1*, *LSH3*, and *PHB*) across five species, aligning with the transcriptomic insights by Shu et al. (Fig. S4) [3]. Therefore, the distribution of auxin and CK is critical for *de novo* shoot formation on pluripotent calli in *C. annuum* ‘Dempsey’ (Fig. 6).

## Conclusion

Our transcriptomic analyses of *C. annuum* ‘Dempsey’ callus and shoot tissue in explants illustrated a regulatory network in which phytohormones significantly impact specific genes to induce *de novo* shoot formation and defense mechanisms. The crux of the comparative transcriptomic analyses is that callus pluripotency may be derived from a balance among mechanisms directing energy towards either developmental processes or photosynthesis, creating the conditions for *de novo* regeneration. The DEGs associated with shoot formation pointed to adaptive actions in response to environmental stresses, and this was observed across five species in a comparative species. The hypoxic condition induced by the lignin barrier created as a defense mechanism induces *de novo* shoot formation in pluripotent callus tissue through *ANT*-mediated primordia growth under oxidative stress resilience. In addition, an auxin-responsive master regulator, *MP*, induces auxin-regulated genes to provide auxin maxima controlled by *PIN1*, promoting primordia growth for *de novo* shoot formation (Fig. 6).

### Electronic supplementary material

Below is the link to the electronic supplementary material.

Supplementary Material 1: Table S1 Primer sequences for quantitative real-time reverse-transcription PCR (qRT-PCR).

Supplementary Material 2: Table S2 Mapping rates of RNA-seq reads to the *C. annuum* ‘Dempsey’ genome.

Supplementary Material 3: Table S3 List of genes involved in defense responses and hypoxia responses in *C. annuum* ‘Dempsey’.

Supplementary Material 4: Data S1 Annotation and differential expression data for *C. annuum* ‘Dempsey’ with four other species for comparison. Data S2 DiVenn 2.0 differential expression data for the callus transcriptomes of *C. annuum* ‘Dempsey’ and four other species. Data S3 DiVenn 2.0 differential expression data for the shoot transcriptomes of *C. annuum* ‘Dempsey’ and four other species. Data S4 Phytohormone-related gene expression in K-means clusters 1, 3, and 5 of *C. annuum* ‘Dempsey’ and four other species.

Supplementary Material 5: Fig. S1 Venn diagrams depicting the commonality of callus and shoot formation-related DEGs across five-species: *C. annuum* ‘Dempsey’, *P. axillaris*, *P. exserta*, *P. integrifolia*, and *A. thaliana*.

Supplementary Material 6: Fig. S2 Chlorophyll content measurement results from ‘Dempsey’ leaf (WT), leaf-derived callus tissue, callus-derived emerging shoot tissue

Supplementary Material 7: Fig. S3 Validation of RNA-seq gene expression by quantitative real-time reverse-transcription PCR (qRT-PCR).

Supplementary Material 8: Fig. S4 Heatmap comparing the gene expression of auxin-related genes for *de novo* shoot formation between the present study and the study by Shu et al. (2022).

## Data Availability

The datasets analyzed during the current study are available in the National Center for Biotechnology Information repository [https://www.ncbi.nlm.nih.gov/bioproject/PRJNA1063381, accession number- PRJNA1063381].

## Abbreviations

ABA: abscisic acid
AMP: adenosine monophosphate
ANT: Aintegumenta
ATP: adenosine triphosphate
BR: brassinosteroid
Callus: leaf explant-derived callus tissue
CK: cytokinin
CKX3: Cytokinin oxidase 3
Cnetplot: gene-concept network plot
CPM: Counts Per Million
DEG: differentially expressed gene
ET: ethylene
FDR: false discovery rate
GA: gibberellic acid
GO: Gene ontology
HD-ZIP III: homeodomain-leucine zipper III
JA: jasmonic acid
LSH3: Light sensitive hypocotyls 3
MP: Monopteros
PIN1: Pin-formed 1
q: probability value
qRT-PCR: quantitative real-time reverse-transcription PCR
ROS: reactive oxygen species
RS: ranking score of NOISeq R package
SA: salicylic acid
SAM: shoot apical meristem
Shoot: callus-driven emerging bud
STM: Shoot meristemless
TMM: Trimmed Mean of M-values
WT: leaf tissue of ‘Dempsey’
WUS: Wuschel
