# Supplementary material for: Elucidating the callus-to-shoot-forming mechanism in Capsicum annuum ‘Dempsey’ through comparative transcriptome analyses
Source: BMC Plant Biol. 2024 May 7;24:367. doi: 10.1186/s12870-024-05033-4 (PMC11075324; doi:10.1186/s12870-024-05033-4)
Supplement: Supplementary file 1 — Supplementary Material 1: Table S1 Primer sequences for quantitative real-time reverse-transcription PCR (qRT-PCR). [file 12870_2024_5033_MOESM1_ESM.docx]

| **Table S1 Primer sequences for quantitative real-time reverse-transcription PCR (qRT-PCR)** | | | |  |
| --- | --- | --- | --- | --- |
| **Gene** | **Gene-ID (Dempsey)** | **Forward (5'-3')** | **Reverse (5'-3')** | **Amplicon (bp)** |
| Actin | CaDEM03G20100 | TCCTAAGGCCAACAGAGAGAA | GCAACATACATAGCTGGAACGT | 72 |
| GAPDH | CaDEM03G33920 | ATGACCACAGTGCACTCGAT | AAGCAGCTCTTCCACCTCTC | 85 |
| ANT | CaDEM04G21840 | GAATTGTGGCATGGCTGGTT | CTCCGTGGCATTACAAGATCTG | 68 |
| MP | CaDEM04G26130 | TCCGGCAGAGTTCAATAGCT | TGTTGGATGTGTTCGATGCA | 91 |
| PIN1 | CaDEM03G42500 | TCCTGGCATGTTCTCACCTA | GATCCTTGCCAGCTTCTTCTC | 80 |
| LSH3 | CaDEM06G33230 | ACGAAGGAATTCAAGGACATGAG | CCTCCATTGGCACTCTCTAGA | 54 |
| PHB | CaDEM02G13220 | GGCTCCAGTGGAATGACAGA | TGGTGATGATGCTCCATTGC | 54 |
