# Supplementary material for: Elucidating the callus-to-shoot-forming mechanism in Capsicum annuum ‘Dempsey’ through comparative transcriptome analyses
Source: BMC Plant Biol. 2024 May 7;24:367. doi: 10.1186/s12870-024-05033-4 (PMC11075324; doi:10.1186/s12870-024-05033-4)
Supplement: Supplementary file 2 — Supplementary Material 2: Table S2 Mapping rates of RNA-seq reads to the C. annuum ‘Dempsey’ genome. [file 12870_2024_5033_MOESM2_ESM.docx]

| **Table S2 Mapping rates of RNA-seq reads to the *C. annuum* 'Dempsey' genome** | | | | | | | | |
| --- | --- | --- | --- | --- | --- | --- | --- | --- |
| **SRA  accession no.** | **Description** | **Data size (KB)** | | **Reads mapped in  pairs (%)** | **Reads mapped in  broken pairs (%)** | **Reads not  mapped (%)** | **Total reads (%)** | **Overall  mapping rate** |
| SRR27483494 | WT1 | 755,800 | 13,201,476 (97.55%) | | 136,083 (1.01%) | 195,907 (1.45%) | 13,533,466 (100%) | 98.55% |
| SRR27483493 | WT2 | 848,054 | 13,803,804 (97.60%) | | 141,917 (1.00%) | 197,161 (1.39%) | 14,142,882 (100%) | 98.61% |
| SRR27483492 | Callus1 | 752,709 | 12,095,808 (97.15%) | | 147,758 (1.19%) | 206,988 (1.66%) | 12,450,554 (100%) | 98.34% |
| SRR27483491 | Callus2 | 705,698 | 11,400,874 (97.25%) | | 134,969 (1.15%) | 187,857 (1.60%) | 11,723,700 (100%) | 98.40% |
| SRR27483490 | Shoot1 | 843,953 | 13,643,458 (97.47%) | | 148,190 (1.06%) | 205,866 (1.47%) | 13,997,514 (100%) | 98.53% |
| SRR27483489 | Shoot2 | 715,585 | 11,596,788 (97.34%) | | 135,081 (1.13%) | 181,557 (1.52%) | 11,913,426 (100%) | 98.48% |
