# Supplementary material for: Elucidating the callus-to-shoot-forming mechanism in Capsicum annuum ‘Dempsey’ through comparative transcriptome analyses
Source: BMC Plant Biol. 2024 May 7;24:367. doi: 10.1186/s12870-024-05033-4 (PMC11075324; doi:10.1186/s12870-024-05033-4)
Supplement: Supplementary file 3 — Supplementary Material 3: Table S3 List of genes involved in defense responses and hypoxia responses in C. annuum ‘Dempsey’. [file 12870_2024_5033_MOESM3_ESM.docx]

| **Table S3 List of genes involved in defense responses and hypoxia response in *C. annuum* 'Dempsey'** | | | | |
| --- | --- | --- | --- | --- |
| **Gene ID (Dempsey)** | **Cluster** | **Gene ID (Arabidopsis)** | **Callus vs. WT (FC)** | **Shoot vs. WT (FC)** |
| **Defensins (8)** |  |  |  |  |
| CaDEM07G00190 | 1 | - | 6.2 | 4.2 |
| CaDEM07G00200 | 1 | AT2G26010 | 9 | 6.4 |
| CaDEM07G00290 | 1 | AT2G31953 | 2.9 | -4.3 |
| CaDEM07G01490 | 1 | AT2G31953 | 4.1 | -2.5 |
| CaDEM07G01510 | 1 | AT2G31953 | 2.2 | 1.2 |
| CaDEM07G01550 | 1 | AT2G31957 | 7.2 | 5.7 |
| CaDEM07G01560 | 1 | - | 5.9 | 4.5 |
| CaDEM12G07160 | 5 | AT2G31953 | 9.3 | 8.8 |
| **CASP-like proteins (2)** |  |  |  |  |
| CaDEM05G03350 | 1 | AT2G39518 | 5.5 | 3 |
| CaDEM05G03420 | 5 | AT2G39530 | 9.1 | 8.9 |
| **Peroxidases (30)** |  |  |  |  |
| CaDEM01G02980 | 1 | AT4G11600 | 2.4 | 1.3 |
| CaDEM01G13340 | 1 | AT4G30170 | 1.6 | 0.8 |
| CaDEM02G20600 | 1 | AT5G05340 | 6.3 | 5.1 |
| CaDEM02G24160 | 1 | AT5G42180 | 2.7 | 2 |
| CaDEM02G25500 | 1 | AT5G66390 | 5.1 | 2.2 |
| CaDEM02G33120 | 1 | AT5G40150 | 0.5 | -0.4 |
| CaDEM03G12920 | 1 | AT4G26010 | 6 | 4.6 |
| CaDEM03G24880 | 1 | AT5G51890 | 3.7 | 1.5 |
| CaDEM03G26840 | 1 | AT5G05340 | 7.4 | 4.9 |
| CaDEM04G16250 | 1 | AT1G71695 | 5.5 | 2.3 |
| CaDEM04G16260 | 1 | AT1G71695 | 3.2 | 1.6 |
| CaDEM05G05880 | 1 | AT1G14550 | 9.6 | 6 |
| CaDEM05G05900 | 1 | AT1G14550 | 7 | 1.8 |
| CaDEM05G05910 | 1 | AT1G14550 | 9.5 | 4.9 |
| CaDEM05G05930 | 1 | AT1G14550 | 4.5 | -0.2 |
| CaDEM05G16490 | 1 | AT5G05340 | 9.6 | 6.7 |
| CaDEM05G20900 | 1 | AT5G05340 | 8.1 | 6.5 |
| CaDEM06G06680 | 1 | AT1G07890 | 0.2 | -1.2 |
| CaDEM06G14870 | 1 | AT4G08390 | 0.5 | 0.1 |
| CaDEM10G19560 | 1 | AT2G37130 | 5.9 | 3.7 |
| CaDEM11G20080 | 1 | AT5G06720 | 6.3 | 4.7 |
| CaDEM01G16180 | 3 | AT5G06720 | -2.9 | 0.5 |
| CaDEM02G21580 | 3 | AT4G21960 | -0.9 | 1 |
| CaDEM02G24680 | 3 | AT4G35000 | 0.1 | 1.3 |
| CaDEM07G18710 | 3 | AT1G05260 | 1 | 2.6 |
| CaDEM02G28730 | 5 | AT4G37530 | 4.9 | 4.5 |
| CaDEM08G19020 | 5 | AT2G41480 | 7.3 | 7 |
| CaDEM09G03420 | 5 | AT2G37130 | 3.3 | 3.5 |
| CaDEM09G04320 | 5 | AT3G09640 | 1.3 | 1.4 |
| CaDEM12G20040 | 5 | AT1G63460 | 0.8 | 0.8 |
