# Supplementary material for: Elucidating the callus-to-shoot-forming mechanism in Capsicum annuum ‘Dempsey’ through comparative transcriptome analyses
Source: BMC Plant Biol. 2024 May 7;24:367. doi: 10.1186/s12870-024-05033-4 (PMC11075324; doi:10.1186/s12870-024-05033-4)
Supplement: Supplementary file 5 — Supplementary Material 5: Fig. S1 Venn diagrams depicting the commonality of callus and shoot formation-related DEGs across five-species: C. annuum ‘Dempsey’, P. axillaris, P. exserta, P. integrifolia, and A. thaliana. [file 12870_2024_5033_MOESM5_ESM.pdf]

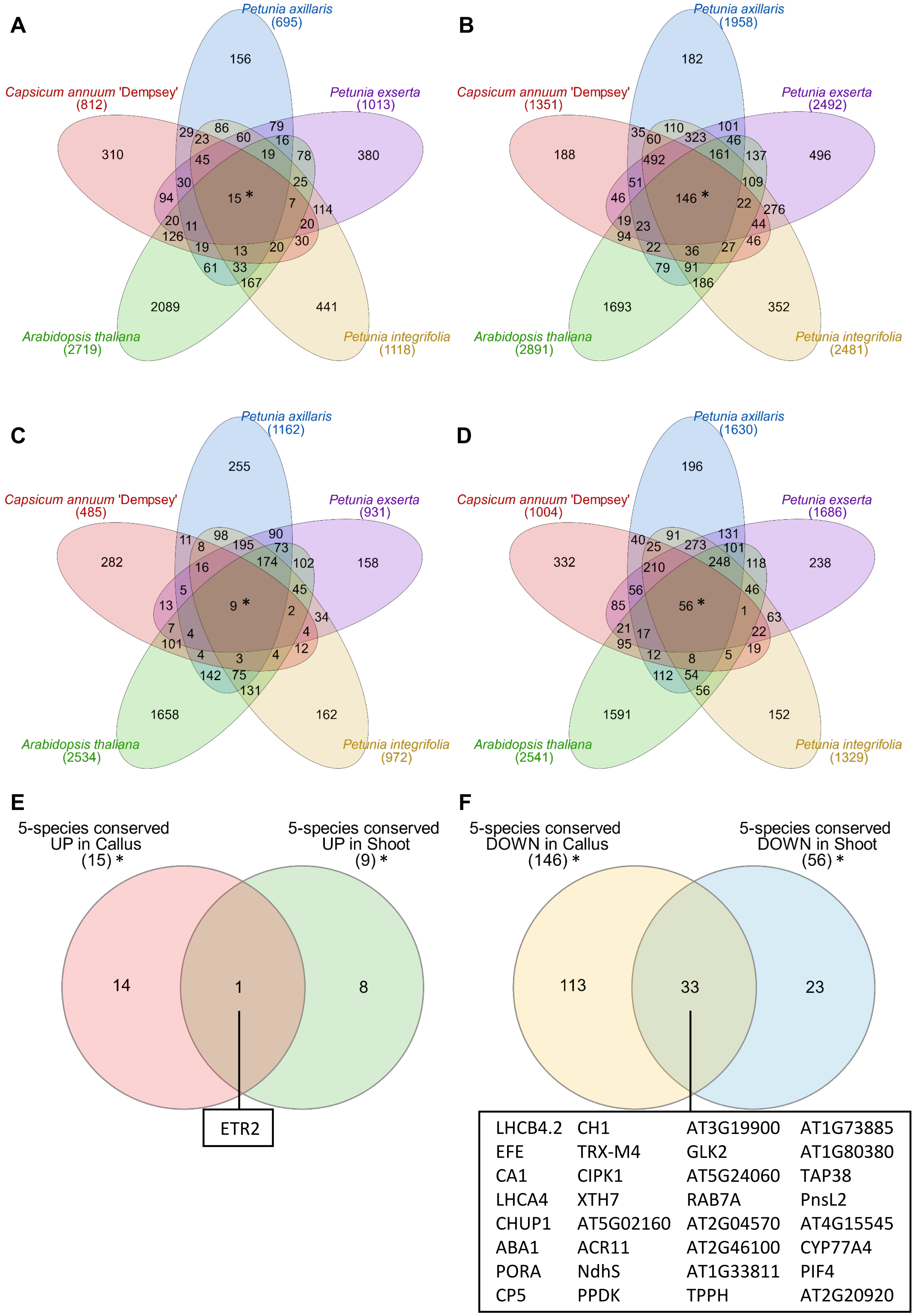


**Fig. S1** Venn diagrams depicting the commonality of callus and shoot formation-related DEGs across five species: *C. annuum* 'Dempsey', *P. axillaris*, *P. exserta*, *P. integrifolia*, and *A. thaliana*. (**A**) A comparison of upregulated genes in the callus tissue; (**B**) A comparison of downregulated genes in the callus tissue; (**C**) A comparison of upregulated genes in the shoot tissue; (**D**) A comparison of downregulated genes in the shoot tissue; (**E**) A comparison of commonly upregulated genes in both callus and shoot tissues across the five species (Callus vs. Shoot); (**F**) A comparison of commonly downregulated genes in both callus and shoot tissues across the five species (Callus vs. Shoot). The boxes below the diagrams contain the genes common to both callus and shoot tissues. The asterisks denote the number of genes with conserved regulation across the five species in callus and shoot tissues in Fig. 4, as further compared in panels E and F.
