# Supplementary material for: Elucidating the callus-to-shoot-forming mechanism in Capsicum annuum ‘Dempsey’ through comparative transcriptome analyses
Source: BMC Plant Biol. 2024 May 7;24:367. doi: 10.1186/s12870-024-05033-4 (PMC11075324; doi:10.1186/s12870-024-05033-4)
Supplement: Supplementary file 6 — Supplementary Material 6: Fig. S2 Chlorophyll content measurement results from ‘Dempsey’ leaf (WT), leaf-derived callus tissue, callus-derived emerging shoot tissue [file 12870_2024_5033_MOESM6_ESM.pdf]

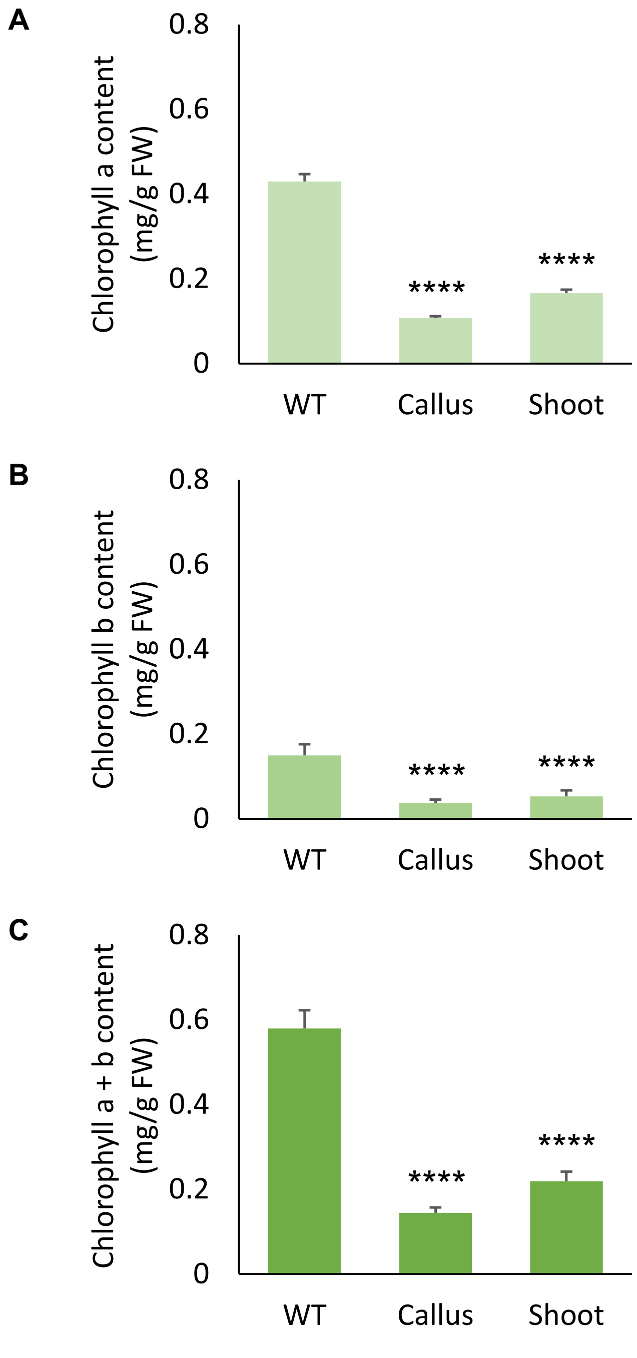


**Fig. S2** Chlorophyll content measurement results (mg/g in fresh weight [FW], $n = 9$, mean ± S.D.) from 'Dempsey' leaf WT (left), leaf-derived callus tissue (middle), callus-derived emerging shoot tissue (right). (**A**) Chlorophyll a content; (**B**) Chlorophyll b content; (**C**) Total chlorophyll (a + b) content. One-tailed Student's T-tests were performed to determine significant differences in chlorophyll content between the groups (Callus vs. WT or Shoot vs. WT), and significance was indicated by asterisks (*$p < 0.05$; **$p < 0.01$; ***$p < 0.001$; ****$p < 0.0001$).
