# Supplementary material for: Elucidating the callus-to-shoot-forming mechanism in Capsicum annuum ‘Dempsey’ through comparative transcriptome analyses
Source: BMC Plant Biol. 2024 May 7;24:367. doi: 10.1186/s12870-024-05033-4 (PMC11075324; doi:10.1186/s12870-024-05033-4)
Supplement: Supplementary file 7 — Supplementary Material 7: Fig. S3 Validation of RNA-seq gene expression by quantitative real-time reverse-transcription PCR (qRT-PCR). [file 12870_2024_5033_MOESM7_ESM.pdf]

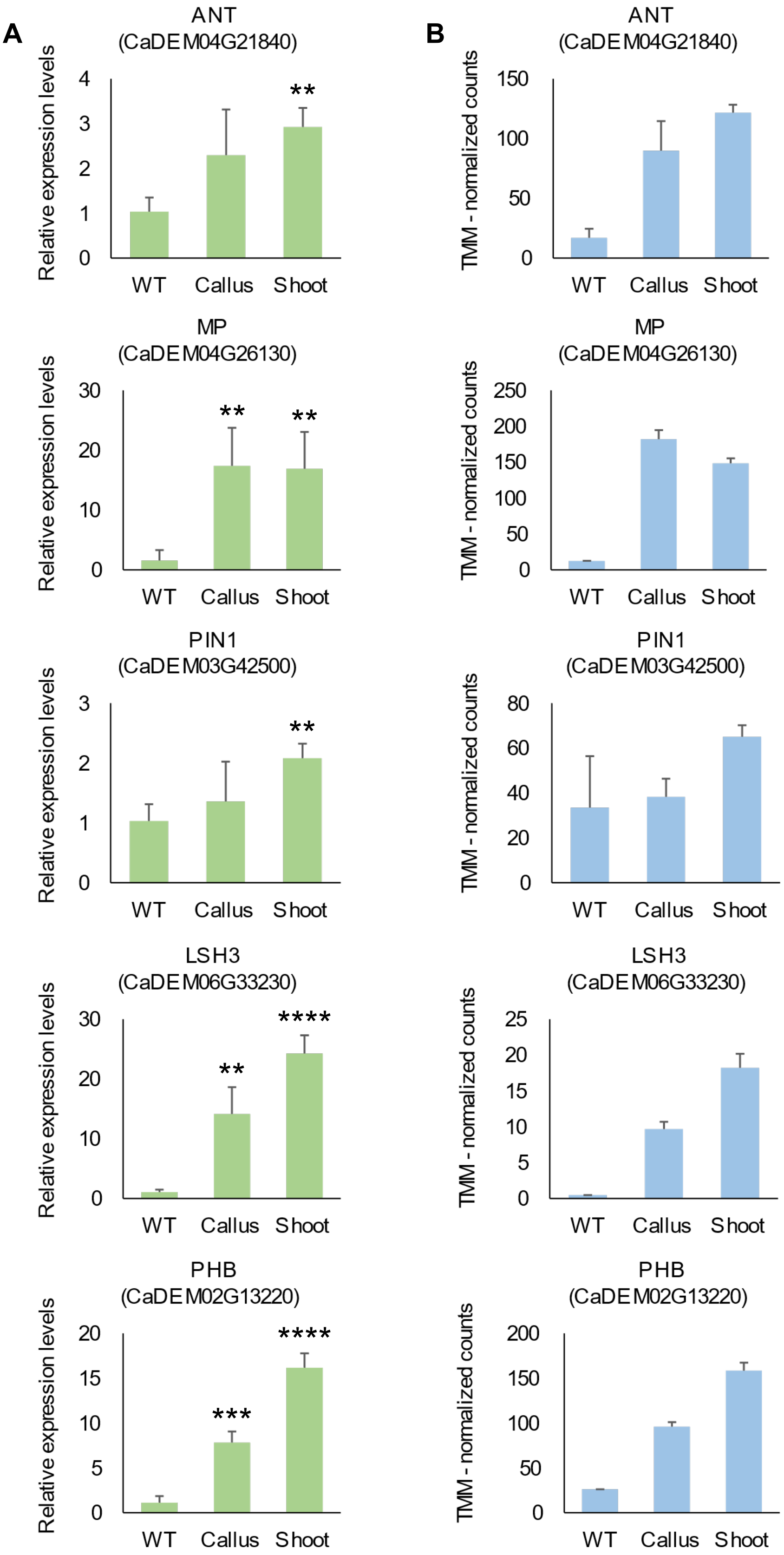

Fig. S3 Validation of RNA-seq gene expression by real-time quantitative reverse transcription PCR (qRT-PCR). (**A**) Relative expression levels of qRT-PCR; (**B**) TMM-normalized counts of RNA-seq. The relative expression level was calculated by the $2^{-\Delta\Delta C_T}$ method (n = 3, mean ± S.D.). The TMM-normalized count was calculated by the Counts Per Million (CPM) using EdgeR (n = 2, mean ± S.D.). One-tailed Student's T-tests were performed to determine significant differences between groups (Callus vs. WT or Shoot vs. WT), and significance was indicated by asterisks (**p* < 0.05; ***p* < 0.01; ****p* < 0.001; *****p* < 0.0001) for qRT-PCR results.
