# Supplementary material for: Elucidating the callus-to-shoot-forming mechanism in Capsicum annuum ‘Dempsey’ through comparative transcriptome analyses
Source: BMC Plant Biol. 2024 May 7;24:367. doi: 10.1186/s12870-024-05033-4 (PMC11075324; doi:10.1186/s12870-024-05033-4)
Supplement: Supplementary file 8 — Supplementary Material 8: Fig. S4 Heatmap comparing the gene expression of auxin-related genes for de novo shoot formation between the present study and the study by Shu et al. (2022). [file 12870_2024_5033_MOESM8_ESM.pdf]

			Present study			Shu et al. (2022)							
			C. annuum 'Dempsey'			*C. baccatum* 'HNUCb226'		*C. baccatum* 'HNUCb81'		*C. chinense* 'HNUCc22'		*C. chinense* 'HNUCc16'	
Gene	*Capsicum annuum* 'Zunla-1' description	Dempsey-ID	log_2 FC-[Callus vs. WT]	log_2 FC-[Shoot vs. WT]	PBC81-ID	log_2 FC-[S2 vs. S1]	log_2 FC-[S3 vs. S1]	log_2 FC-[S2 vs. S1]	log_2 FC-[S3 vs. S1]	log_2 FC-[S2 vs. S1]	log_2 FC-[S3 vs. S1]	log_2 FC-[S2 vs. S1]	log_2 FC-[S3 vs. S1]
ANT	AP2-like ethylene-responsive transcription factor ANT	CaDEM04G21840	2.47	2.95	CQW23_05338	3.13	3.76	1.63	1.73	3.39	3.30	3.27	3.03
MP	auxin response factor 5	CaDEM04G26130	3.95	3.65	CQW23_11194	4.98	6.43	3.01	3.34	3.72	3.94	4.25	3.78
PIN1	probable auxin efflux carrier component 1c	CaDEM03G42500	0.31	1.11	CQW23_11571	0.62	0.31	0.76	0.44	1.09	0.54	1.48	1.00
LSH3	protein LIGHT-DEPENDENT SHORT HYPOCOTYLS 4-like	CaDEM06G33230	8.30	9.24	CQW23_07316	0.23	1.02	0.88	0.78	1.29	1.53	1.07	1.92
PHB	homeobox-leucine zipper protein ATHB-14 isoform X2	CaDEM02G13220	1.89	2.62	CQW23_04063	1.39	2.46	0.99	1.10	1.33	1.77	2.30	1.99

log2 FC
2
-2

Fig. S4 Heatmap comparing the gene expression of auxin-related genes for *de novo* shoot formation between the present study and the study by Shu et al. (2022). RNA-seq data were analyzed to identify comparative DEGs in *C. annuum* 'Dempsey' (red), *C. baccatum* 'HNUCb226' and 'HNUCb81' (grey), and *C. chinense* 'HNUCc22' and 'HNUCc16' (navy). The annotation for the gene descriptions was based on *Capsicum annuum* 'Zunla-1', and homologous genes in *C. baccatum* and *C. chinense* were denoted using the *C. baccatum* 'PBC81' annotation. The heat intensity color scale represents a log_2 fold change (log_2|FC|). A detailed annotation is provided in Data S1.
